# Global, regional, and 204 countries burden of disease attributed to secondhand smoke-related COPD, 1990–2021: A secondary data analysis of the Global Burden of Disease Study 2021

**DOI:** 10.18332/tid/230940

**Published:** 2026-09-12

**Authors:** Qian Huang, Lewen Fu, Sajid Ali, Shengtian Hou, Kang Wang, Ruifeng Li, Bo Ao, Youliang Huang

**Affiliations:** 1 Department of Respiratory Medicine, Beijing Tian Tan Hospital, Capital Medical University, Beijing, China; 2 School of Health Policy and Management, Peking Union Medical College (Chinese Academy of Medical Sciences), Beijing, China; 3 Department of Information Sciences, University of Education, Multan, Pakistan; 4 Faculty of Health and Wellness, City University of Macau, Macau, China; 5 School of Management, Capital Normal University, Beijing, China; 6 School of Management, Beijing University of Chinese Medicine, Beijing, China; 7 Department of Pediatrics, China-Japan Friendship Hospital, Beijing, China; 8 National Institute of Chinese Medicine Development and Strategy, Beijing University of Chinese Medicine, Beijing, China

**Keywords:** chronic obstructive pulmonary disease, secondhand smoke, disease burden, EAPC, ARIMA model

## Abstract

**INTRODUCTION:**

Chronic obstructive pulmonary disease (COPD) imposes a significant economic burden on global public health systems. This secondary data analysis aims to estimate the disease burden of COPD attributable to secondhand smoke exposure from 1990 to 2021.

**METHODS:**

Data on mortality, age-standardized mortality rate (ASMR), and age-standardized disability-adjusted life years rate (ASDR) for COPD attributable to secondhand smoke exposure were collected from the Global Burden of Disease (GBD 2021) database. The percentage changes in estimated annual percentage change, number of deaths, and number of disability-adjusted life years were calculated. The associations between ASMR and sociodemographic index (SDI), as well as between ASDR and SDI, were also investigated. The autoregressive integrated moving average time series model was used to project future trends through 2031.

**RESULTS:**

Between 1990 and 2021, the number of deaths worldwide attributable to secondhand smoke exposure leading to COPD increased. ASMR decreased from 11.36 (95% UI: 4.52–18.55) to 5.87 (95% UI: 2.29–9.72), while ASDR decreased from 234.01 (95% UI: 93.58–380.47) to 120.94 (95% UI: 47.2–197.93). In 2021, China had the highest mortality (117387.96; 95% UI: 47776.16–195915.33), while Papua New Guinea had the highest ASMR (31.63; 95% UI: 11.96–54.96) and ASDR (597.51; 95% UI: 226.75–1036.97). The decline in COPD mortality and ASDR was greater among females than among males. SDI exhibited a nonlinear association with both ASMR and ASDR, with SDI=0.42 representing the inflection point for this relationship. Furthermore, the predictive model estimates that ASMR and ASDR attributable to secondhand smoke exposure for COPD will continue declining through 2031.

**CONCLUSIONS:**

Globally, both ASMR and ASDR for COPD attributable to secondhand smoke exposure have shown a declining trend, which is projected to continue. However, the magnitude of decline and absolute disease burden vary across income-heterogeneous countries. There is an urgent need to strengthen awareness interventions targeting vulnerable populations and enforce indoor smoke-free policies to prevent further harm.

## INTRODUCTION

Chronic obstructive pulmonary disease (COPD) is a chronic airway disease primarily characterized by persistent respiratory symptoms and airflow limitation^1^. It is typically closely associated with environmental factors such as long-term smoking and prolonged exposure to harmful gases or particulate matter, as well as individual factors including genetic susceptibility^2^. Patients with COPD commonly experience symptoms such as dyspnea, cough, sputum production, wheezing, and fatigue, which progressively worsen as the disease advances^3^. The pathogenesis of COPD is complex and multifaceted, involving various pathophysiological processes including systemic inflammation, oxidative stress, cellular senescence, and dysregulated autophagy, with interrelated mechanisms^4^. The Global Burden of Disease (GBD) study indicates that in 2021, COPD affected 21.34 million people globally, resulting in approximately 79.78 million disability-adjusted life years (DALYs) lost^5^. By 2021, COPD ranked as the third leading cause of mortality worldwide, accounting for about 5% of all global mortality^6,7^. The burden of COPD varies across regions due to differences in population size and socioeconomic development. East and South Asia bear a disproportionately high burden of COPD, exceeding the global average^8^. Furthermore, similar to many major noncommunicable diseases, the burden of COPD is projected to increase in most regions worldwide as populations age^9^.

Secondhand smoke (SHS) refers to the smoke produced by burning tobacco that non-smokers are forced to inhale in an environment, primarily composed of mainstream smoke and sidestream smoke^10^. Although exposure to SHS involves inhaling less smoke than active smoking, individuals chronically exposed to SHS still inhale significant amounts of harmful substances and suffer damage comparable to that of active smokers. SHS contains over 7000 chemicals, at least 250 of which are known to be harmful to humans, and more than 69 are confirmed carcinogens. Prolonged exposure to SHS severely damages human health, increasing adults’ risk of cardiovascular disease, cancer, and respiratory illnesses^11^. Research indicates that SHS exposure is a significant and preventable risk factor for COPD, closely linked to rising COPD incidence rates^12,13^. Tobacco smoke in SHS binds to the MRI protein, reducing the activity and function of mucosal-associated invariant T cells in the lungs. This accelerates lung function decline and increases susceptibility to COPD^14^.

COPD is characterized by high incidence, high disability rates, and high mortality, causing severe physical health damage and socioeconomic burdens worldwide^15^. Despite significant progress in global tobacco control, SHS exposure remains a prevalent public health issue. Concurrently, the shifting dynamics of macro public health factors, particularly rapid population aging and the heterogeneous implementation of tobacco control policies, significantly modulate the long-term trends of this disease burden. To our knowledge, while existing GBD studies have examined the overall disease burden of COPD and the impact of its primary risk factors, no study has yet addressed recent changes in the COPD disease burden attributable to SHS exposure. A secondary analysis using GBD 2021 data was conducted to estimate the global, regional, and national COPD burden attributable to SHS exposure across 204 countries and territories from 1990 to 2021. Comparative temporal trends were estimated in the COPD burden attributable to SHS exposure globally, regionally, and across 204 countries and territories from 1990 to 2021. This study aimed to depict the patterns and comparative temporal trends of the COPD disease burden associated with SHS exposure across varying geographical areas and demographic groups. Furthermore, the correlation between COPD and the sociodemographic index (SDI) was explored, and future trends over the next decade were projected. These findings aim to enhance global awareness of SHS exposure and provide empirical epidemiological evidence to assist governments, public health agencies, and policymakers in optimizing targeted tobacco control policies and COPD prevention strategies.

## METHODS

### Study population and data collection

The epidemiological data on COPD attributable to SHS exposure from 1990 to 2021 used in this study were sourced from the GBD database^16^. This database integrates epidemiological, demographic, and health-related data globally, covering mortality rates, DALYs for 369 diseases and injuries across 204 countries and regions, as well as exposure and effects of 87 risk factors. COPD mortality and morbidity data are primarily derived from vital registration systems, hospital registries, and epidemiological surveys, while SHS exposure data are gathered from household surveys. Data are reported across multiple dimensions, including cause of death, age, sex, year, and geographical location. The Global Health Data Exchange GBD platform was accessed, and GBD mortality and DALYs for COPD attributable to SHS exposure from 1990 to 2021 were downloaded. Given varying COPD risk factors across populations, gender- and age-specific estimates for COPD were also provided. Furthermore, to ensure comparability across countries, regions, and time periods, the study employed standardized data based on the age structure of the World Health Organization Standard Population^17^, providing a unified reference system for international comparative studies of population health indicators. As the data used in this study contained no identifiable information, ethical review was not required, and informed consent was waived.

### Definition of evaluation indicators

Adult COPD patients aged ≥25 years were categorized by gender and further subdivided into 15 age groups (25–29, 30–34, 35–39, 40–44, 45–49, 50–54, 55–59, 60–64, 65–69, 70–74, 75–79, 80–84, 85–89, 90–94, ≥95 years). The SDI is a composite indicator reflecting a country’s lagging per capita income distribution among females aged <25 years, average years of schooling, and fertility rate^18,19^. The data for these core components are systematically collected and estimated by the GBD covariates team using multinational survey programs, national censuses, and administrative records. Countries and regions are classified into five SDI categories (high-middle, high, low-middle, low, and middle). The SDI ranges from 0 to 1, where 0 represents the lowest development level and 1 represents the highest development level. Estimates based on 2021 GBD data use age-standardized rates per 100000 population.

### Definition of COPD

According to the Global Initiative for Chronic Obstructive Lung Disease (GOLD) classification, COPD is defined as a measured value of <0.7 FEV1/ FVC (forced expiratory volume in one second/forced vital capacity)^20-22^. COPD severity is also graded according to the GOLD classification. When FEV1 scores are 80% of normal, 50–79% of normal, or ≤50% of normal, the condition is characterized as mild (I), moderate (II), or severe (III) or very severe combined (IV). COPD encompasses diseases coded as J41, J42, J43, J44, and J47 in the International Classification of Diseases (ICD) 10th and 49th editions (ICD-10 and ICD-49th), as well as disease 496 in the ICD 9th edition.

### Statistical analysis

Considering the impact of raw data and measurement bias, the data were normalized based on the standard population age structure, providing a unified reference system for comparative studies of population health indicators internationally^17^. This study selected mortality rates, DALYs, years of life lost (YLL), and years lived with disability (YLD) to evaluate the disease burden of COPD attributable to SHS exposure. All estimates employed Monte Carlo simulations to integrate age-specific data and report 95% uncertainty intervals (UI). Estimated annual percentage change (EAPC) values for age-standardized mortality rate (ASMR), age-standardized DALYs rate (ASDR), age-standardized years of life lost (ASYLL), and age-standardized years lived with disability (ASYLD) attributable to SHS exposure were calculated using linear regression models for the period 1990–2021. To visualize geographical variations in SHS-attributable COPD burden by ASMR and ASDR in 2021, maps of COPD attributable to SHS exposure were generated. The percentage change in SHS-attributable COPD from 1990 to 2021 was defined as [(2021 value - 1990 value)/1990 value]×100%. The relationship between the COPD burden attributable to SHS exposure, stratified by location and year, and the SDI was evaluated using Spearman’s rank correlation test. The correlation coefficient (ρ) ranges from -1 to 1, where ρ >0 indicates a positive association, ρ <0 indicates a negative association, and values closer to 1 or -1 represent stronger correlation strength. The ARIMA model was employed because of its established efficacy and robustness in modeling long-term, non-stationary chronic disease historical trends and smooth dependencies. Optimal parameters for the ARIMA model were selected based on the Akaike information criterion (AIC) and Bayesian information criterion (BIC). The *forecast()* function was used to project the COPD disease burden in Asia from 2022 to 2031. All statistical analyses were performed using R software (version 4.4.2), and a two-sided p<0.05 was defined as the statistical threshold for significance.

## RESULTS

### Overall impact of SHS exposure on the disease burden of COPD

Supplementary file Figure S1 illustrates the global trends in ASMR, ASDR, ASYLD, and ASYLL for COPD attributable to SHS exposure from 1990 to 2021. During that time, ASMR, ASDR, ASYLD, and ASYLL for COPD attributable to SHS exposure showed a declining trend among women. For men, except for ASYLD, both ASMR and ASDR for COPD attributed to SHS exposure showed a declining trend, while ASYLL also decreased. Regarding changes in ASYLD for men, the decline was relatively gradual in regions with high SDI, while other regions exhibited a pattern of initial increase followed by a decrease.

The study also presents EAPC analyses of ASMR, ASDR, ASYLL, and ASYLD attributable to SHS exposure-related COPD from 1990 to 2021, categorized by SDI and 21 regions (Table 1). Globally, the overall trend for all four indicators of COPD attributable to SHS exposure shows a decline. Middle SDI regions exhibited the most pronounced declines in ASMR, ASDR, and ASYLL, with a slower decrease in ASYLD. High-middle SDI regions followed. Eastern Europe, East Asia, and Tropical Latin America demonstrated more pronounced downward trends than other regions. Detailed findings are presented in Supplementary file Tables S1–S4.

**Table 1:** Estimated average percentage change (EAPC) of ASMR, ASDR, ASYLL, and ASYLD from SHS exposure-related COPD from 1990 to 2021, by SDI and 21 Regions, a secondary data analysis based on the Global Burden of Disease Study 2021

*Location*	*Deaths* *EAPC (95% CI)*	*DALYs* *EAPC (95% CI)*	*ASYLL* *EAPC (95% CI)*	*ASYLD* *EAPC (95% CI)*
**Global SDI regions**	-2.54 (-2.67 – -2.41)	-2.5 (-2.61 – -2.39)	-0.53 (-0.59 – -0.46)	-2.83 (-2.96 – -2.70)
High	-2.37 (-2.46 – -2.28)	-2 (-2.06 – -1.95)	-1.06 (-1.15 – -0.98)	-2.37 (-2.45 – -2.29)
High-middle	-3.62 (-3.86 – -3.37)	-3.45 (-3.65 – -3.25)	-0.36 (-0.4 – -0.31)	-4.01 (-4.25 – -3.78)
Middle	-3.66 (-3.83 – -3.48)	-3.64 (-3.8 – -3.48)	-0.79 (-0.84 – -0.75)	-4.04 (-4.21 – -3.86)
Low-middle	-0.85 (-0.96 – -0.74)	-1.08 (-1.16 – -0.99)	-0.62 (-0.76 – -0.49)	-1.14 (-1.23 – -1.06)
Low	-0.78 (-0.94 – -0.62)	-1.07 (-1.19 – -0.96)	-0.65 (-0.83 – -0.47)	-1.14 (-1.26 – -1.02)
**Regions**				
Andean Latin America	-2.7 (-2.79 – -2.61)	-2.71 (-2.81 – -2.62)	-1.68 (-1.87 – -1.49)	-2.98 (-3.07 – -2.89)
Australasia	-3.22 (-3.45 – -2.99)	-3.2 (-3.39 – -3.00)	-2.41 (-2.55 – -2.28)	-3.38 (-3.64 – -3.12)
Caribbean	-0.79 (-1.04 – -0.54)	-0.72 (-0.96 – -0.47)	-0.63 (-0.83 – -0.43)	-0.73 (-0.99 – -0.47)
Central Asia	-2.3 (-2.56 – -2.04)	-2.19 (-2.43 – -1.95)	-0.13 (-0.18 – -0.08)	-2.58 (-2.86 – -2.30)
Central Europe	-2.43 (-2.56 – -2.29)	-1.95 (-2.06 – -1.84)	-0.62 (-0.66 – -0.58)	-2.33 (-2.47 – -2.2)
Central Latin America	-2.91 (-3.07 – -2.76)	-2.81 (-2.96 – -2.67)	-1.6 (-1.69 – -1.51)	-3.04 (-3.2 – -2.88)
Central Sub-Saharan Africa	-1.53 (-1.62 – -1.44)	-1.44 (-1.53 – -1.35)	-0.54 (-0.6 – -0.47)	-1.63 (-1.72 – -1.54)
East Asia	-4.61 (-4.82 – -4.41)	-4.5 (-4.69 – -4.32)	-0.75 (-0.83 – -0.66)	-4.96 (-5.17 – -4.75)
Eastern Europe	-5.66 (-6.03 – -5.28)	-4.57 (-4.9 – -4.24)	-1.79 (-2.01 – -1.57)	-5.35 (-5.74 – -4.96)
Eastern Sub-Saharan Africa	-2.43 (-2.54 – -2.33)	-2.13 (-2.23 – -2.03)	-1 (-1.08 – -0.92)	-2.39 (-2.5 – -2.29)
High-income Asia Pacific	-4.11 (-4.29 – -3.93)	-3.07 (-3.14 – -3.00)	-1.68 (-1.84 – -1.51)	-4.18 (-4.35 – -4.00)
High-income North America	-1.17 (-1.33 – -1.00)	-1.29 (-1.42 – -1.16)	-1.27 (-1.42 – -1.12)	-1.29 (-1.42 – -1.16)
North Africa and the Middle East	-1.17 (-1.34 – -0.99)	-1.14 (-1.24 – -1.05)	0.08 (0.01–0.16)	-1.47 (-1.6 – -1.34)
Oceania	-0.61 (-0.68 – -0.53)	-0.65 (-0.72 – -0.58)	-0.15 (-0.2 – -0.09)	-0.69 (-0.76 – -0.62)
South Asia	-1.1 (-1.24 – -0.96)	-1.32 (-1.42 – -1.21)	-0.83 (-1.02 – -0.64)	-1.39 (-1.49 – -1.29)
Southeast Asia	-1.72 (-1.85 – -1.59)	-1.63 (-1.73 – -1.52)	-0.52 (-0.58 – -0.46)	-1.82 (-1.94 – -1.71)
Southern Latin America	-1.47 (-1.76 – -1.17)	-1.59 (-1.83 – -1.35)	-0.97 (-1.07 – -0.86)	-1.66 (-1.92 – -1.40)
Southern Sub-Saharan Africa	-1.41 (-1.77 – -1.05)	-1.17 (-1.45 – -0.89)	-1.03 (-1.09 – -0.98)	-1.21 (-1.57 – -0.85)
Tropical Latin America	-4.36 (-4.67 – -4.04)	-4.04 (-4.35 – -3.73)	-2.31 (-2.43 – -2.19)	-4.27 (-4.6 – -3.93)
Western Europe	-2.16 (-2.23 – -2.08)	-1.93 (-1.98 – -1.88)	-1.18 (-1.28 – -1.08)	-2.2 (-2.27 – -2.13)

ASMR: age-standardized mortality rate per 100000 population. ASDR: age-standardized DALYs rate per 100000 population. ASYLL: age-standardized years of life lost. ASYLD: age-standardized years of life disabled. COPD: chronic obstructive pulmonary disease. SHS: secondhand smoke. SDI: sociodemographic index. EAPC: estimated annual percentage change. CI: confidence interval.

### Global distribution of COPD disease burden attributable to SHS exposure

**Figure 1:**
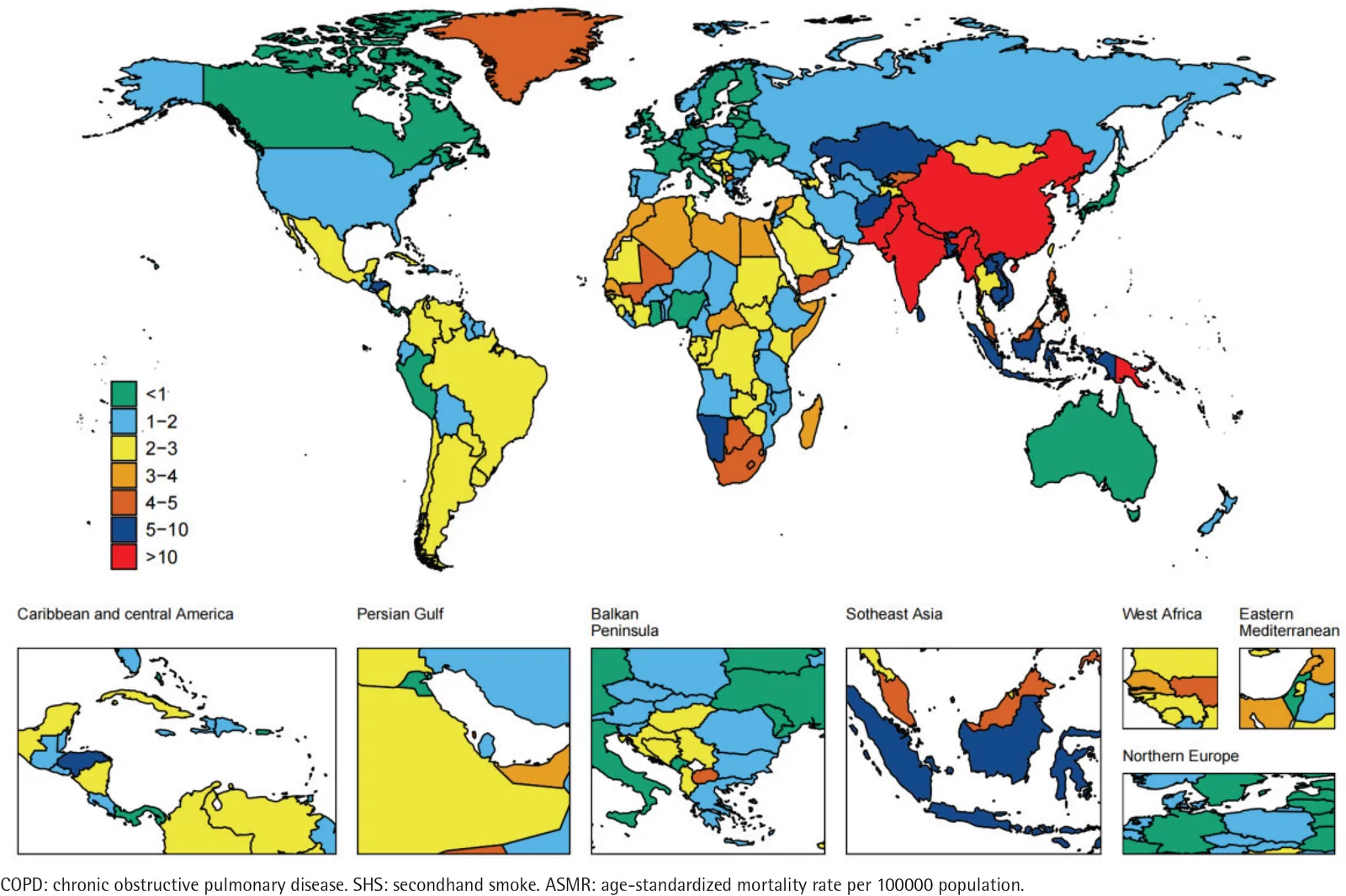
Global distribution of ASMR for COPD attributable to SHS exposure in 2021, a secondary data analysis across 204 countries and territories based on the Global Burden of Disease Study 2021

**Figure 2:**
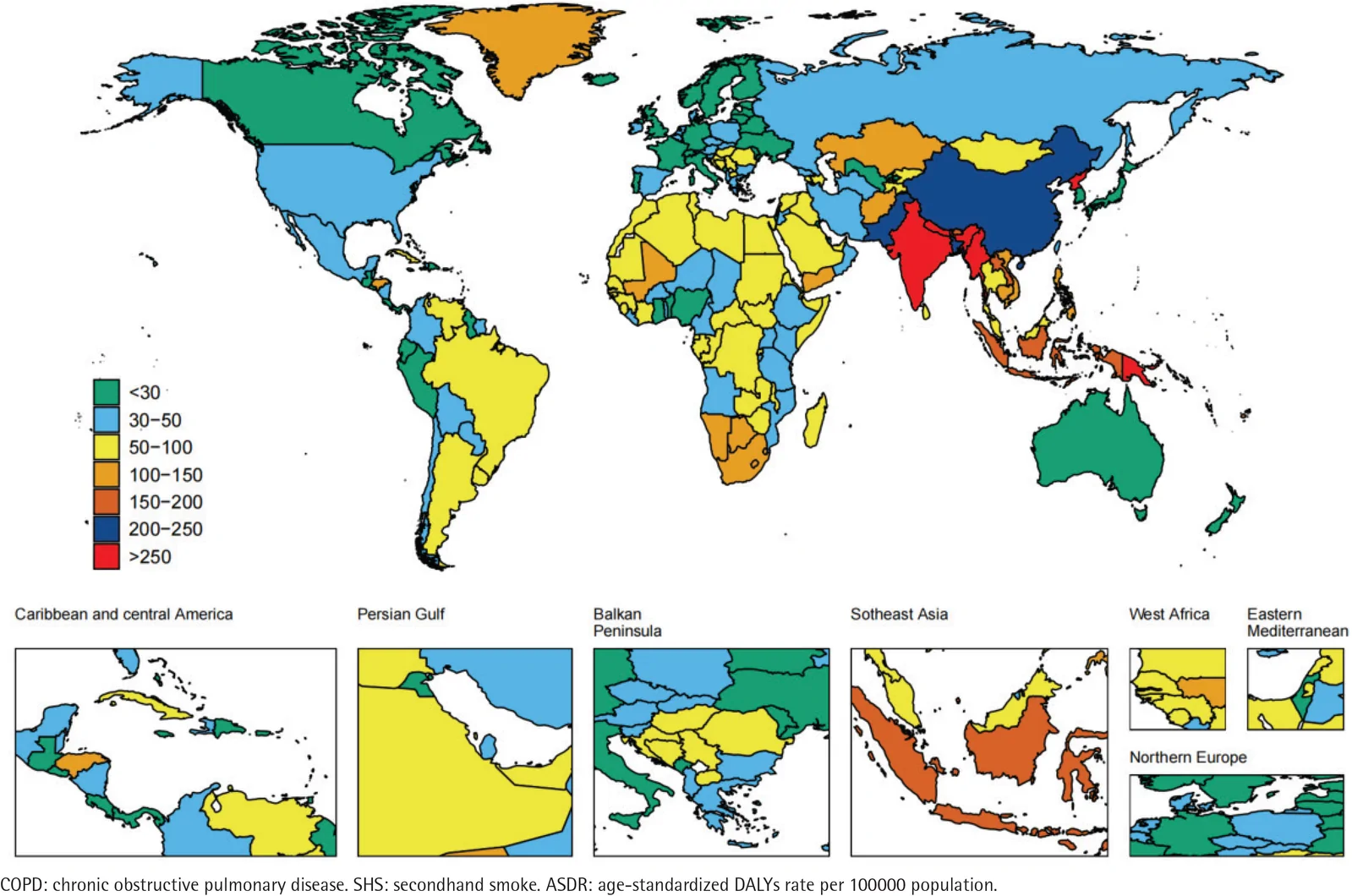
Global distribution of ASDR for COPD attributable to SHS exposure in 2021, a secondary data analysis across 204 countries and territories based on the Global Burden of Disease Study 2021

Among 204 countries and regions, China (117387.96; 95% UI: 47776.16–195915.33) and India (75614.28; 95% UI: 28341.18–124842.16) recorded the highest COPD mortality attributable to SHS exposure in 2021, with all other countries and regions reporting fewer than 10000 mortality (Supplementary file Table S5). In 2021, China (2182250.92; 95% UI: 876774.91–3556235.66) and India (1669829.85; 95% UI: 619318.64–2735802.85) also recorded the highest number of DALYs. However, the highest ASMR for COPD mortality attributable to SHS exposure in 2021 occurred in Papua New Guinea (31.63; 95% UI: 11.96–54.96), followed by Kiribati (21.72; 95% UI: 8.13–39.25), followed by Nepal (20; 95% UI: 7.61–34.79) (Figure 1; and Supplementary file Table S5). The highest ASDR in 2021 were also observed in Papua New Guinea (597.51; 95% UI: 226.75–1036.97), Kiribati [424.09; (95% UI: 163.63–745.01), and Nepal (377.88; 95% UI: 144.39–655.49) (Figure 2; and Supplementary file Table S5).

**Figure 3:**
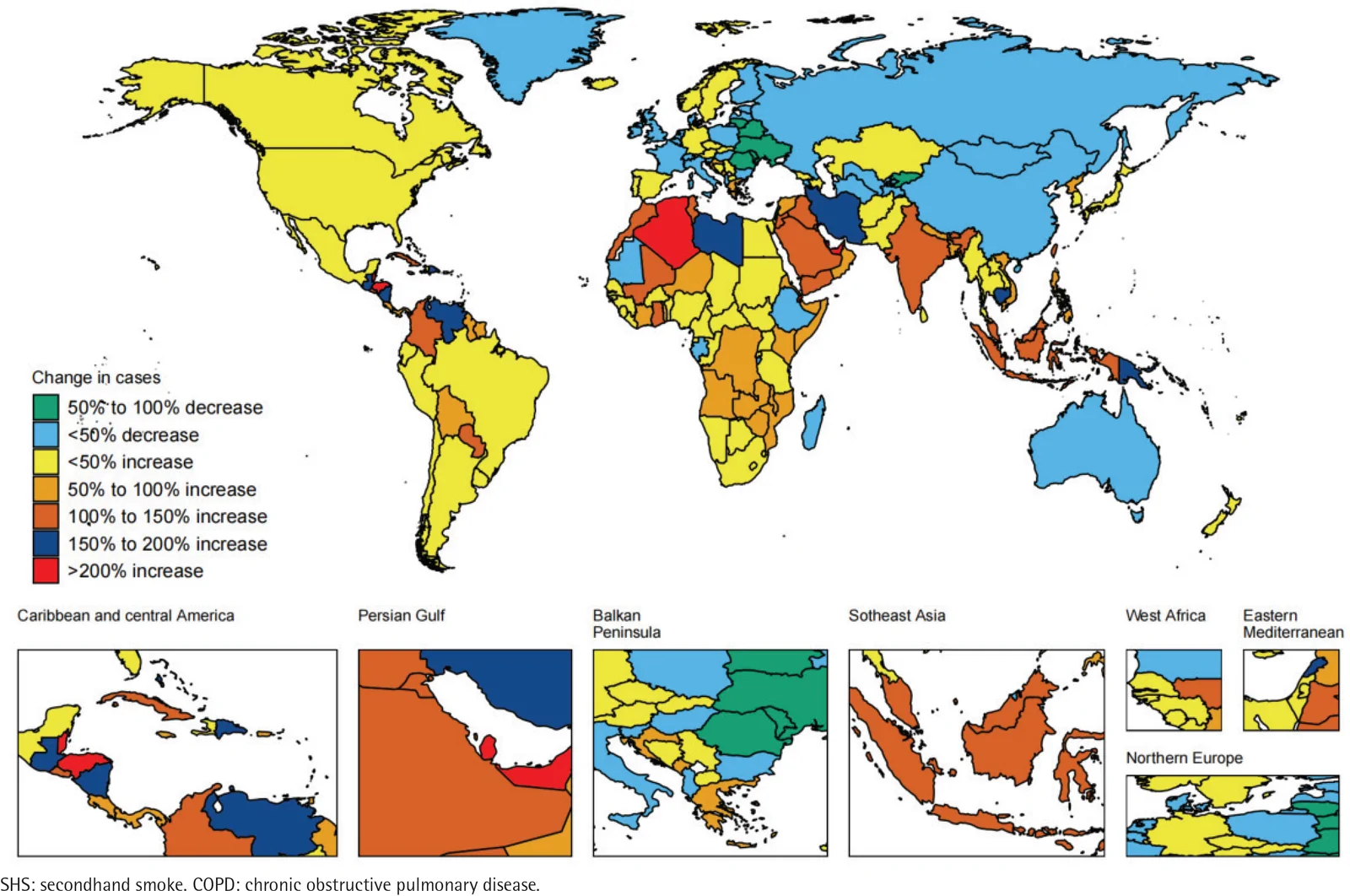
Proportion of change in mortality due to COPD attributable to SHS exposure from 1990 to 2021, a secondary data analysis across 204 countries and territories based on the Global Burden of Disease Study 2021

**Figure 4:**
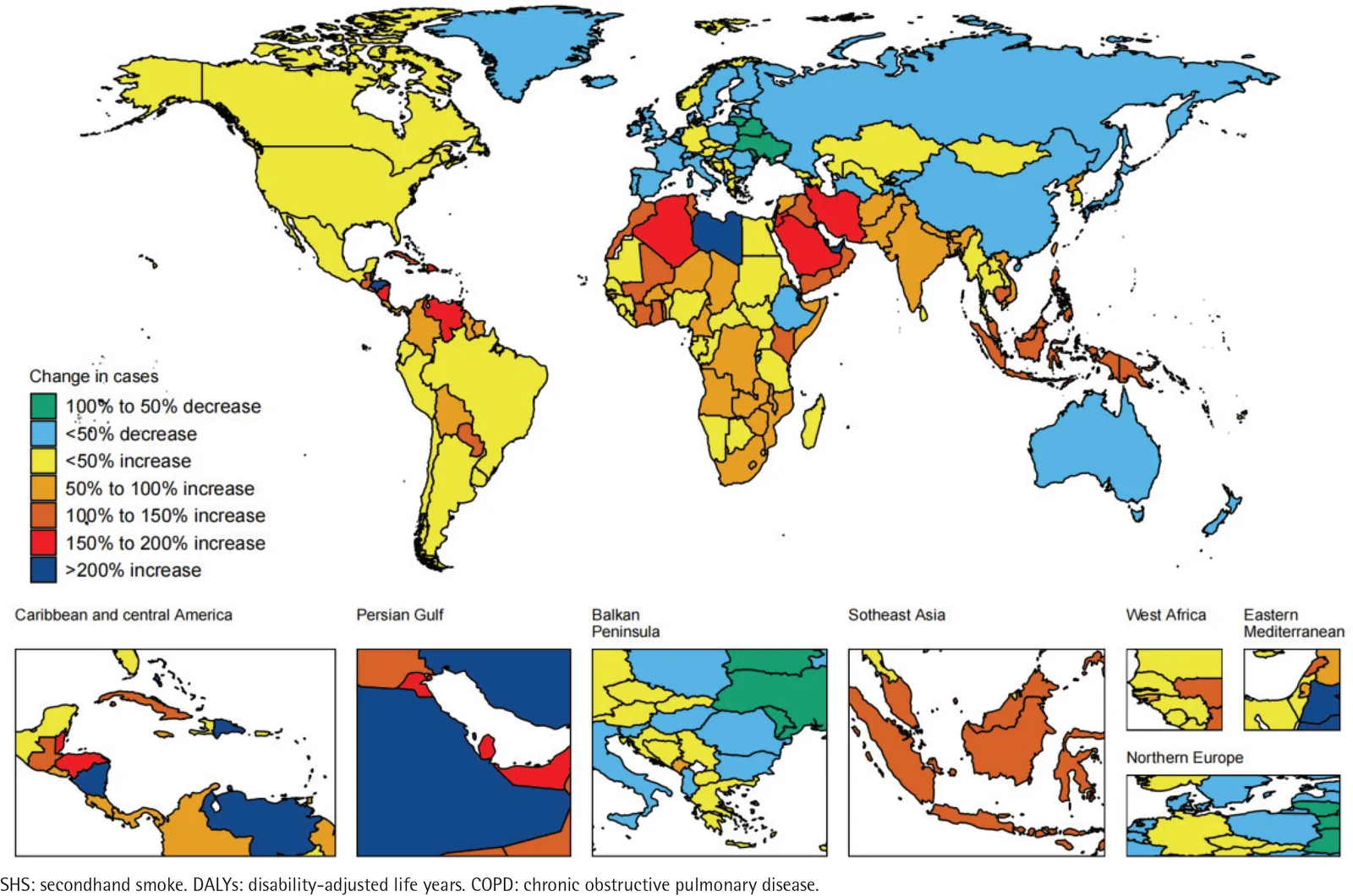
Proportion of change in DALYs due to COPD attributable to SHS exposure from 1990 to 2021, a secondary data analysis across 204 countries and territories based on the Global Burden of Disease Study 2021

Notably, between 1990 and 2021, the countries and regions experiencing the fastest growth in COPD deaths attributable to SHS exposure were Belize (267.92%) and Honduras (246.15%), while the countries and regions with the fastest decline were Ukraine (-85.95%) and Belarus (-83.88%) (Figure 3; and Supplementary file Table S5). The countries and regions with the fastest growth in DALYs were Qatar (435.65%) and the United Arab Emirates (395.08%), while the fastest decline was observed in Ukraine (-81.53%) and Belarus (-73.13%) (Figure 4; and Supplementary file Table S5).

### Correlation analysis between COPD disease burden from SHS exposure and SDI

In 2021, countries and regions with middle SDI recorded the highest number of COPD deaths attributable to SHS exposure (104890.54; 95% UI: 42778.94–172646.77), and the highest number of DALYs (2132844.46; 95% UI: 869137.14–3478022.09). Countries and regions with high SDI had the lowest number of mortalities (14985.48; 95% UI: 5966.28–24,623.12) and the lowest number of DALYs [359565.29; 95% UI: 144011.77–585561.32) attributable to SHS exposure-related COPD. Low SDI countries and regions had the second-lowest mortality (16190.95 (95% UI: 6161.47–26927.79) and the second-lowest number of DALYs (392852.57; 95% UI: 148021.95–647490.07). Compared to 1990, only the high-middle SDI region saw decreases in both mortality (-7.69%) and DALYs (-12.99%). Mortality and DALYs increased across all other SDI regions, with the largest increases observed in the low-middle SDI region: 87.04% for mortality and 71.07% for DALYs. In 2021, the low-middle SDI region had the highest ASMR (11.57; 95% UI: 4.44–19.25) and ASDR (230.16; 95% UI: 88.18–381.63), while the high SDI region had the lowest ASMR (1.16; 95% UI: 0.46–1.94) and ASDR (31.79; 95% UI: 12.45–52.6) (Supplementary file Table S1).

The relationship between SDI attributable to SHS and the corresponding ASMR and ASDR for COPD across 21 GBD regions from 1990 to 2021 was examined (Supplementary file Figure S2). Overall, the ASMR for SHS-related COPD exhibited a nonlinear relationship with SDI from 1990 to 2021, showing a significant downward trend as SDI increased (ρ= -0.517, p<0.001). The ASDR of COPD attributable to SHS exposure also exhibited the same relationship with SDI from 1990 to 2021 (ρ= -0.503, p<0.001). When SDI was <0.42, both ASMR and ASDR for COPD attributable to SHS positively correlated with SDI. Between 0.42 and 0.7, a negative correlation with a significant downward trend emerged. Above 0.7, a negative correlation persisted, but the decline slowed considerably. From 1990 to 2021, ASMR and ASDR exceeded projections in East Asia, Oceania, and South Asia. ASMR and ASDR fell below projections in Central Sub-Saharan Africa, Western Sub-Saharan Africa, North Africa and the Middle East, the Caribbean, Central Latin America, Tropical Latin America, Andean Latin America, and Australasia. Additionally, ASMR in Central Asia was below projections. Eastern Sub-Saharan Africa exhibited higher-than-expected ASMR in the early period, though it declined later (Supplementary file Figure S2A). Eastern Sub-Saharan Africa and Eastern Europe showed higher-than-expected ASDR in the early period, which was better controlled in the later period (Figure S2B).

### ARIMA model predictions for the Global COPD disease burden from 2022 to 2031

This study employed time series modeling to conduct dynamic analysis and projections of the core indicators ASMR and ASDR, stratified by gender. AIC and BIC were utilized to select optimal parameters for the ARIMA models, with Supplementary file Table S6 detailing the selected parameters. Residuals from all models passed the Ljung-Box test (p>0.05), indicating satisfactory data fit. Supplementary file Table S7 presents projected ASMR and ASDR by gender from 2022 to 2031. Both ASMR and ASDR for COPD attributable to SHS exposure exhibit a declining trend during this period (Supplementary file Figure S3). By 2031, the global ASMR for COPD attributable to SHS exposure is projected to decrease to 4.59 (95% UI: 2.96–6.22), the male ASMR will decrease to 5.35 (95% UI: 4.2–6.5), and the female ASMR will decrease to 4.97 (95% UI: 2.31–7.64). The global ASDR for COPD attributable to SHS exposure will decrease to 115.79 (95% UI: 73.29–158.28), with the male ASDR decreasing to 103.83 (95% UI: 80.72–126.94), while the female ASDR will decrease to 109.02 (95% UI: 64.23–153.81).

## DISCUSSION

This study aims to assess the global burden of COPD attributable to SHS exposure. From 1990 to 2021, both COPD mortality and DALYs attributable to SHS exposure increased, while ASMR and ASDR showed a declining trend. Compared to 1990, the absolute mortality attributable to SHS exposure experienced a notable expansion, whereas the corresponding ASMR was nearly halved globally over the three-decade period. The burden of COPD attributable to SHS exposure remained disproportionately higher among women, albeit with a more accelerated decline rate between 1990 and 2021. Additionally, SDI exhibited a nonlinear association with both ASMR and ASDR for COPD attributable to SHS exposure, characterized by an initial positive correlation in lower socioeconomic strata followed by a persistent negative correlation as regional development progressed beyond a critical developmental threshold. Over the next decade, both the ASMR and ASDR of COPD attributable to SHS exposure are projected to decline. Although there is currently no complete cure for COPD, it can be prevented and avoided by reducing the impact of risk factors^23^. Therefore, it is essential to precisely quantify the health impacts of COPD attributable to SHS exposure, identify high-risk populations and regions, and comprehensively assess its global disease burden.

This study found that from 1990 to 2021, both the number of deaths and the number of DALYs attributable to SHS exposure for COPD increased, while the ASMR and ASDR decreased. The reasons for this trend include the potential increase in absolute COPD mortality and DALYs driven primarily by global population growth and rapid population aging, alongside the refinement of diagnostic and detection methods contributing to this phenomenon. However, improvements in medical care and diagnostic techniques can reduce ASMR and ASDR for COPD attributable to SHS exposure. Nationwide enhancements in cultural education and medical literacy can also significantly alter COPD risk factors such as age and gender, thereby influencing ASMR and ASDR. Despite a global downward trend in the overall disease burden, substantial variations in the magnitude of decline across different regions were observed. When regions are categorized by SDI, the most pronounced declines in ASMR and ASDR occur in middle SDI regions, followed by high-middle SDI regions. Middle SDI regions are typically undergoing rapid industrialization and urbanization, potentially reflecting insufficient early emphasis on regulating smoking environments during development phases. As SDI increases, local urbanization processes typically bring substantial improvements in healthcare accessibility and medical infrastructure, while the alignment between rising economic levels and stricter tobacco control policies drives noticeable improvements in public health. Compared to high SDI regions, Middle SDI regions may have higher levels of SHS exposure. However, compared to low SDI regions, middle SDI regions possess stronger material foundations for improvement. Consequently, the disease burden of COPD attributable to SHS exposure shows significant improvement in middle SDI regions.

After adjusting for age standardization, ASMR and ASDR reveal patterns in disease risk evolution, yet significant heterogeneity persists in disease burden patterns across countries and regions. China and India, due to their large populations and status as developing nations, exhibit the highest numbers of deaths and DALYs for COPD attributable to SHS exposure, highlighting the need to consider the sustainability of healthcare systems. Although countries like Papua New Guinea, Kiribati, and Nepal appear to have relatively low numbers of deaths and DALYs, their ASMR and ASDR are actually the highest, indicating a higher relative risk than other nations. We believe these countries and regions may suffer from inadequate restrictions on tobacco environments and insufficient public health education, resulting in significant SHS exposure in indoor settings such as public places and homes. Additionally, this study emphasized changes in absolute burden by calculating percentage changes in mortality and DALYs. Belize and Honduras experienced the fastest growth in mortality, while Qatar and the United Arab Emirates saw the most rapid increase in DALYs. This partially reflects potentially substantial pressure and burden on the healthcare systems of these countries and regions. Learning from countries and regions with low ASMR and low ASDR regarding tobacco control policies and disease prevention strategies is essential for preventing and avoiding COPD attributable to SHS exposure^24^. Drawing insights from healthcare management systems and funding strategies in regions with low percentages of mortality and low percentages of DALYs holds significant implications for allocating healthcare resources and addressing national disease burdens^25^.

The study also found significant differences between males and females in ASMR, ASDR, ASRYLL, and ASRYLD for COPD attributable to SHS exposure. In 1990, all assessment metrics for women exceeded those for men. Between 1990 and 2021, the decline in these metrics was also greater for women than for men. This disparity may stem from women constituting a smaller proportion of active smokers. Consequently, most of the ASMR and ASDR indicators among women are attributable to SHS exposure, whereas those among men are predominantly driven by active smoking harm^26^. The severity of SHS exposure correlates with the number and habits of active smokers, and studies indicate that most SHS exposure occurs in household settings^27-29^. Therefore, as awareness of SHS hazards increases and tobacco control policies strengthen, the decline in male active smokers may lead to a significant reduction in female victims of SHS exposure. To systematically protect this vulnerable cohort of non-smoking women, targeted public health strategies – such as community-led smoke-free home programs and gender-specific educational interventions focusing on household secondhand smoke exposure – should be actively prioritized. Additionally, due to COPD’s long latency period and the time required for SHS-induced diseases to manifest^30^, victims exposed before the age of 25 years are unlikely to show clinical signs. This explains why this study exclusively included participants aged ≥25 years.

At the regional level, both the ASMR and ASDR for COPD attributable to SHS exposure exhibit nonlinear relationships with the SDI. Socioeconomic status is independently associated with COPD and correlates with the level of SHS exposure^31^. The SDI is a composite indicator measuring per capita income, years of schooling, and total fertility rate. Regardless of SDI level, ASMR and ASDR in East Asia, Oceania, and South Asia exceeded expectations. This indicates a heavier burden of COPD attributable to SHS in these regions, underscoring the need for enhanced tobacco control policies and public awareness campaigns on SHS hazards. In regions with low socioeconomic development, the SDI positively correlated with both ASMR and ASDR. Eastern Sub-Saharan Africa exhibits higher-than-expected ASMR and ASDR, while Eastern Europe shows higher-than-expected ASDR. As regional development surpasses this critical socioeconomic threshold, the disease burden indicators in these areas begin to fall below expected levels. This may stem from widespread smoking prevalence, insufficient awareness of SHS management, and underdeveloped healthcare systems in Eastern Sub-Saharan Africa and Eastern Europe at lower SDI levels^32^.

Predictive analysis indicates that the ASMR and ASDR for COPD attributable to SHS exposure will continue to decline globally for all genders from 2022 to 2031. This suggests that over time, the number of COPD cases attributable to SHS exposure may decrease, with this trend being more pronounced among males. By 2031, ASMR and ASDR for COPD attributable to SHS exposure are projected to decline due to the anticipated implementation and enforcement of several measures. First, strengthening management of healthy environments by establishing and promoting healthy, smoke-free, and clean settings^33^. Strictly enforcing policies prohibiting smoking in enclosed public spaces and advocating against smoking in homes to minimize airborne smoke persistence^34^. Second, raising public awareness about smoking in indoor environments and disseminating information on the hazards of SHS exposure will reduce smoke pollution and the number of passive smokers^35,36^. Third, enhancing medical detection, prevention, and treatment technologies while optimizing the allocation of healthcare resources will minimize new infections and mortality.

### Strengths and limitations

This study is the first analysis to apply the latest GBD data to focus on COPD attributable to SHS exposure. Our findings reflect the epidemiological status of COPD attributable to SHS exposure, providing a theoretical reference for policy formulation and resource allocation by government departments and public health institutions.

However, our study has several limitations. First, given the cross-sectional and ecological design of the GBD database, our analysis was conducted at the population and country level rather than at the individual level. Thus, the possibility of ecological fallacy cannot be excluded, and the observed associations cannot be directly interpreted as reflecting individual-level risk or establishing definitive causation. Second, data collection constraints may introduce misclassification bias. COPD detection relies heavily on regional diagnostic capabilities, and SHS exposure metrics are partly derived from self-reported surveys, potentially introducing recall bias. Third, despite the GBD framework’s adjustments, the influence of residual confounding from unmeasured environmental or socioeconomic factors cannot be entirely eliminated. Finally, while the ARIMA model is highly robust for smoothing historical time-series data and forecasting future trends, potential methodological covariate biases may still persist in the projections.

## CONCLUSIONS

The global incidence and mortality rates of COPD attributable to SHS exposure show a declining trend, with projections indicating potential further reductions. However, the magnitude of decline and absolute disease burden vary across countries with differing income levels. To mitigate the burden of COPD attributable to SHS, public health strategies should prioritize populations with limited awareness of prevention measures and strengthen the implementation of policy frameworks to curtail indoor smoking.

## Supplementary Material



## Data Availability

The data supporting this research are available from the authors on reasonable request.
