# Supplementary material for: Global, regional, and 204 countries burden of disease attributed to secondhand smoke-related COPD, 1990–2021: A secondary data analysis of the Global Burden of Disease Study 2021
Source: Tob Induc Dis. 2026 Sep 12;24:10.18332/tid/230940. doi: 10.18332/tid/230940 (PMC13589477; doi:10.18332/tid/230940)
Supplement: Supplementary file 1 [file TID-24-164-s1.pdf]

Tobacco Induced Diseases

Supplementary file

© 2026 Huang Q. et al.

DOI:
10.18332/tid/230940

The content has been provided by the author(s) and has not been reviewed, verified, or endorsed by European Publishing. It may not have undergone peer review. The views, opinions, and recommendations expressed are solely those of the author(s) and do not necessarily reflect the position of European Publishing. European Publishing accepts no responsibility or liability for any consequences arising from the use of, or reliance on, this content.

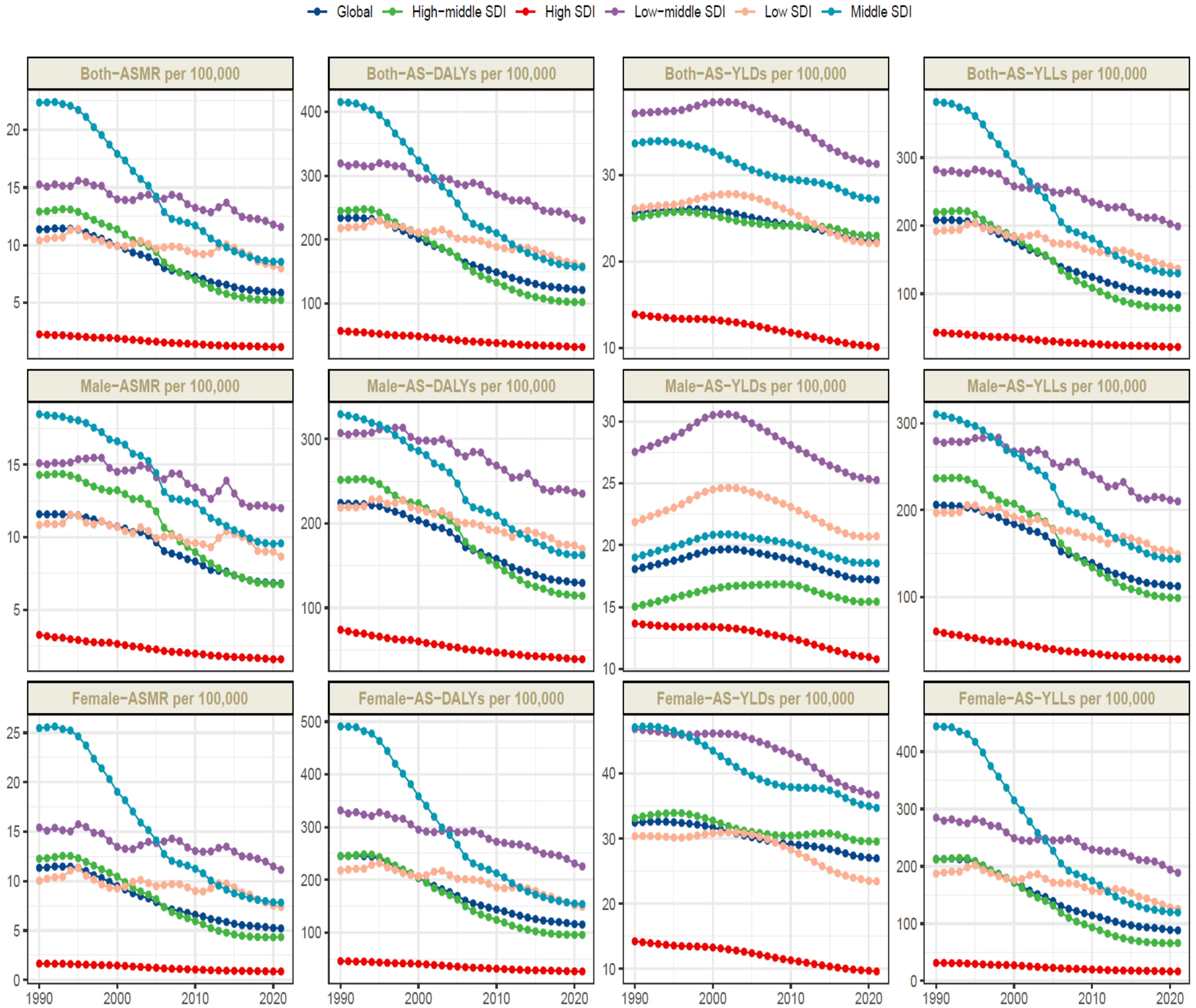

Supplementary Figure S1. ASMR, ASDR, AS-YLLs, and AS-YLDs for COPD attributable to SHS exposure from 1990 to 2021, classified by SDI. A secondary data analysis across 204 countries and territories based on the Global Burden of Disease Study 2021. ASMR - age-standardized mortality rates, ASDR - age-standardized disability-adjusted life year rates, AS-YLLs - age-standardized years of life lost, AS-YLDs - age-standardized years lived with disability, COPD - chronic obstructive pulmonary disease, SHS - secondhand smoke, SDI - socio-demographic index.

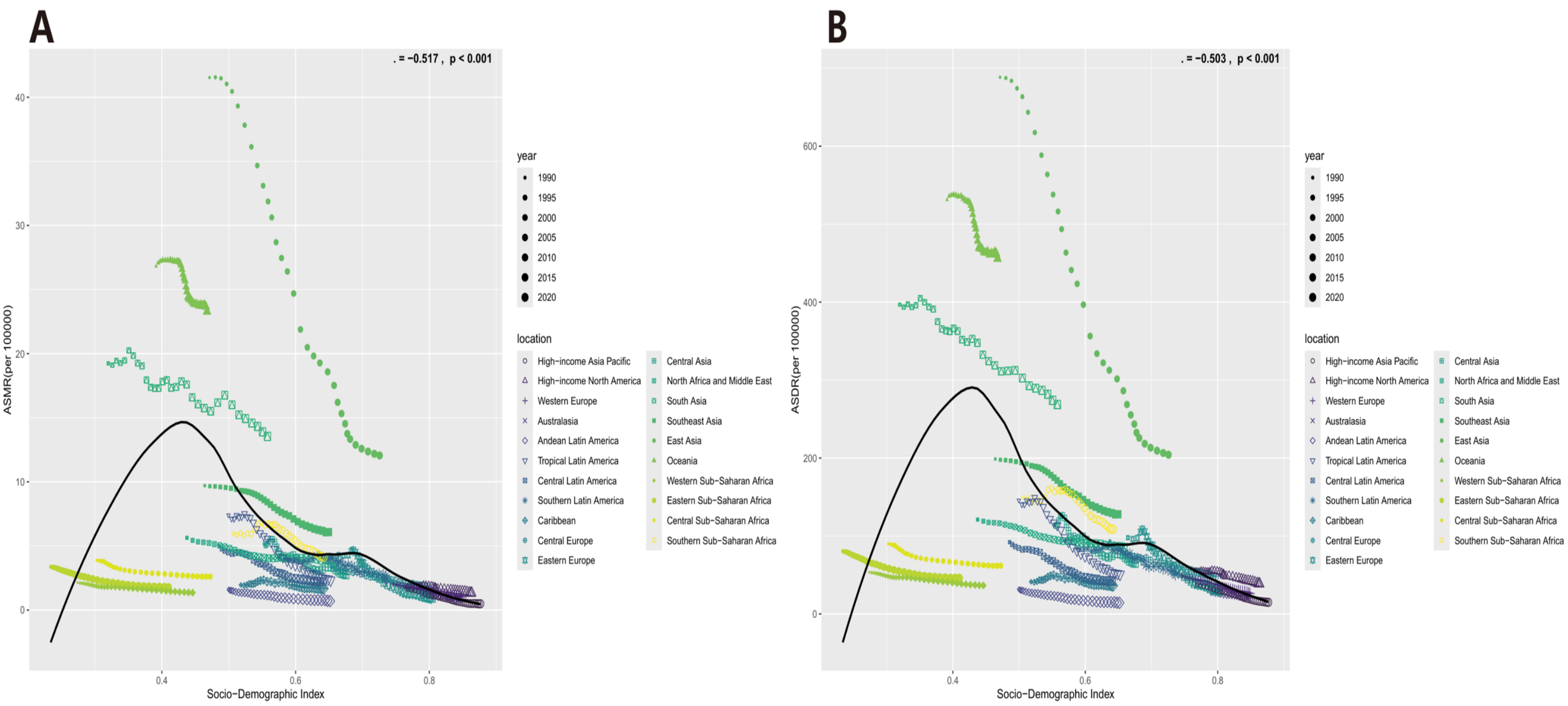

Supplementary Figure S2. Relationship between the ASMR and SDI, as well as between SDI and ASDR, attributable to SHS exposure in 21 GBD regions from 1990 to 2021. Projected values based on SDI and disease rates across all locations are shown as black lines. A secondary data analysis across 204 countries and territories based on the Global Burden of Disease Study 2021. ASMR - age-standardized mortality rates, ASDR - age-standardized disability-adjusted life year rates, GBD - global burden of disease, SHS - secondhand smoke, SDI - socio-demographic index.

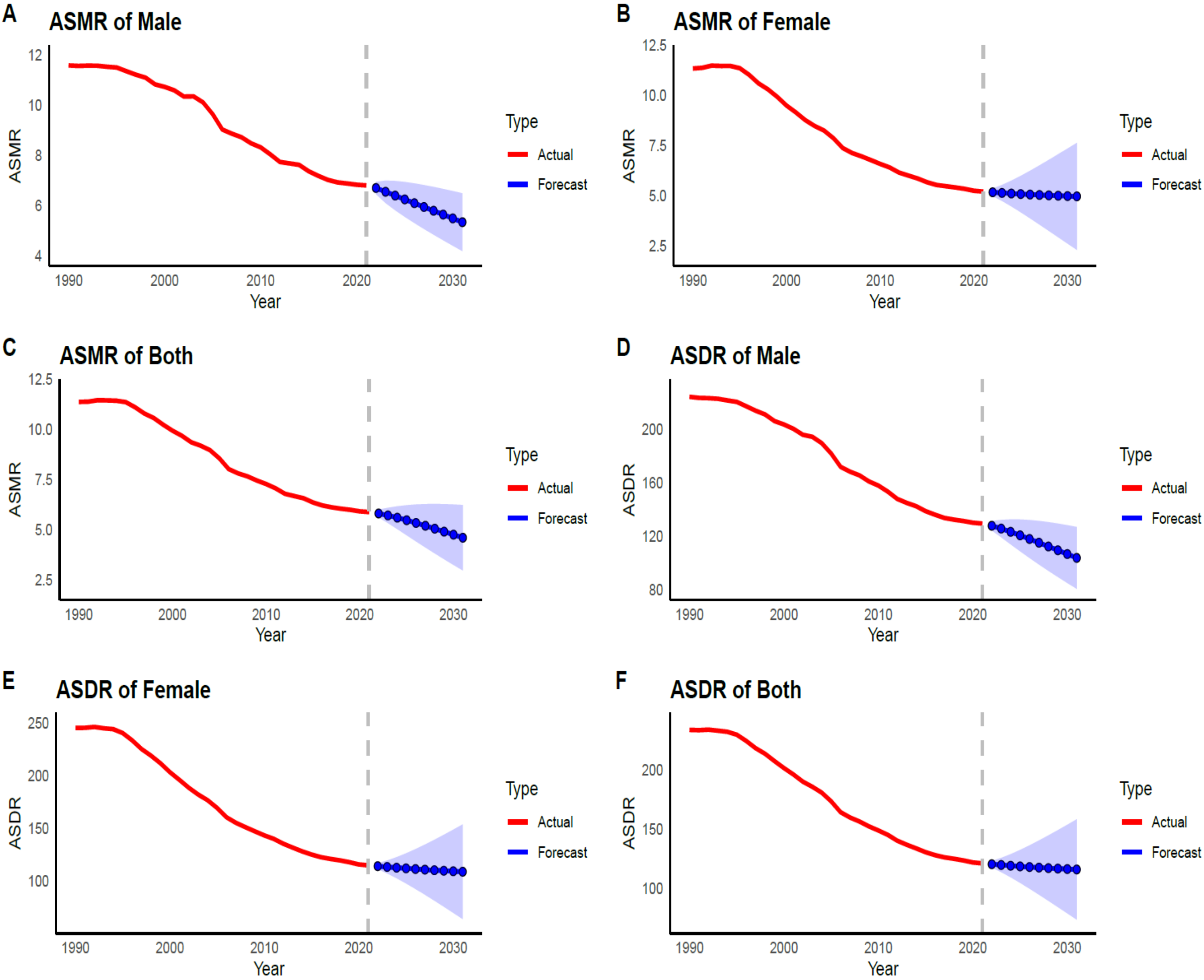

Supplementary Figure S3. Projected disease burden of COPD attributable to SHS exposure for the general population, males, and females, 2022 – 2031. A trend projection analysis across 204 countries and territories based on the Global Burden of Disease Study 2021. COPD - chronic obstructive pulmonary disease, SHS - secondhand smoke.

Supplementary Table S1. Global, by SDI, regional number of deaths, ASMR, and EAPC changes for 1990 and 2021

Location	Number 1990	ASR 1990	Number 2021	ASR 2021	EAPC_95%CI
Global	217147.47 (85577.18-352540.52)	11.36 (4.52-18.55)	266124.7 (106054.27-434215.02)	5.87 (2.29-9.72)	-2.54 (-2.67 to -2.41)
High SDI	14027.56 (5560.33-22967.23)	2.26 (0.89-3.71)	14985.48 (5966.28-24623.12)	1.16 (0.46-1.94)	-2.37 (-2.46 to -2.28)
High-middle SDI	60005.48 (23224.98-98578.03)	12.91 (5.07-21.23)	55393.03 (22274.34-92140.47)	5.23 (2.05-8.86)	-3.62 (-3.86 to -3.37)
Middle SDI	93617.59 (37196.54-151477.55)	22.37 (8.91-36.65)	104890.54 (42778.94-172646.77)	8.55 (3.32-14.35)	-3.66 (-3.83 to -3.48)
Low-middle SDI	39856.78 (15604.77-66712.3)	15.27 (6.07-25.52)	74546.14 (29434.4-123115.06)	11.57 (4.44-19.25)	-0.85 (-0.96 to -0.74)
Low SDI	9539.98 (3800.37-16007.75)	10.4 (4.12-17.65)	16190.95 (6161.47-26927.79)	7.98 (3.02-13.49)	-0.78 (-0.94 to -0.62)
Andean Latin America	155 (61.53-256.69)	1.62 (0.62-2.73)	215 (80.12-369.05)	0.7 (0.25-1.25)	-2.7 (-2.79 to -2.61)
Australasia	298.45 (116.32-496.21)	2.29 (0.84-4)	287.77 (111.23-493.22)	0.9 (0.33-1.62)	-3.22 (-3.45 to -2.99)
Caribbean	252.4 (102.01-413.35)	1.95 (0.76-3.25)	507.74 (201.08-857.91)	1.68 (0.63-2.87)	-0.79 (-1.04 to -0.54)
Central Asia	1191.74 (478.93-1932.57)	5.19 (2.08-8.49)	1027.67 (401.14-1684.33)	2.75 (1.07-4.58)	-2.3 (-2.56 to -2.04)
Central Europe	3071.25 (1245.77-4959.29)	4.03 (1.61-6.56)	2284.87 (911.21-3816.8)	1.78 (0.69-2.94)	-2.43 (-2.56 to -2.29)
Central Latin America	1805.88 (737.37-2905.59)	4.97 (1.98-8.06)	2831.26 (1073.58-4626.91)	2.2 (0.87-3.68)	-2.91 (-3.07 to -2.76)
Central Sub-Saharan Africa	349.41 (129.5-666.06)	3.88 (1.34-7.71)	566.19 (216.57-1120.96)	2.61 (0.9-5.47)	-1.53 (-1.62 to -1.44)
East Asia	125727.46 (49091.75-205805.46)	41.54 (16.25-68.81)	120731.24 (49030.26-200484.49)	12.06 (4.64-20.66)	-4.61 (-4.82 to -4.41)
Eastern Europe	6084.98 (2502.52-9706.17)	4.33 (1.71-7.1)	1935.57 (757.13-3190.37)	0.98 (0.37-1.7)	-5.66 (-6.03 to -5.28)
Eastern Sub-Saharan Africa	1086.76 (436.33-1844.37)	3.37 (1.32-5.83)	1325.44 (518.6-2314.93)	1.75 (0.69-3.09)	-2.43 (-2.54 to -2.33)
High-income Asia Pacific	1524.59 (591.61-2523.34)	1.58 (0.59-2.68)	1744.15 (658.1-2969.58)	0.49 (0.18-0.87)	-4.11 (-4.29 to -3.93)
High-income North America	3634.78 (1442.53-6037.14)	1.81 (0.7-3.04)	5043.88 (1966.67-8330.45)	1.32 (0.5-2.29)	-1.17 (-1.33 to -1)
North Africa and Middle East	3940.46 (1562.05-6586.85)	5.64 (2.21-9.64)	7325.96 (2889.15-12015.13)	3.81 (1.45-6.51)	-1.17 (-1.34 to -0.99)
Oceania	295.05 (108.36-512.79)	26.81 (9.75-47.01)	668.65 (262.57-1089.5)	23.29 (8.87-39.65)	-0.61 (-0.68 to -0.53)
South Asia	45186.57 (18379.49-75065.19)	19.24 (7.67-32.26)	89203.11 (34242.14-146419.48)	13.55 (5.07-22.7)	-1.1 (-1.24 to -0.96)
Southeast Asia	10387.77 (4183.68-16954.2)	9.7 (3.85-16.1)	17591.52 (6763.02-28798.19)	6.08 (2.3-10.01)	-1.72 (-1.85 to -1.59)
Southern Latin America	927.65 (365.82-1490.39)	3.95 (1.55-6.44)	1229.7 (483.04-2021.84)	2.45 (0.93-4.12)	-1.47 (-1.76 to -1.17)

Southern Sub-Saharan Africa	741.67 (299.75-1211.7)	5.95 (2.32-10.18)	1103.74 (438.39-1779.93)	4.11 (1.58-6.84)	-1.41 (-1.77 to -1.05)
Tropical Latin America	2965.97 (1216.09-4748.32)	7.4 (2.97-12.08)	3172.4 (1225.38-5265.27)	2.32 (0.88-4.05)	-4.36 (-4.67 to -4.04)
Western Europe	6661.64 (2634.31-10849.85)	2.03 (0.79-3.35)	6152.44 (2427.21-10259.91)	1.02 (0.39-1.72)	-2.16 (-2.23 to -2.08)
Western Sub-Saharan Africa	857.98 (348.67-1474.34)	2.14 (0.85-3.67)	1176.39 (458.96-2003.09)	1.35 (0.52-2.32)	-1.44 (-1.48 to -1.39)

SDI - socio-demographic index, ASMR - age-standardized mortality rates, EAPC - estimated annual percentage change.

Supplementary Table S2. Global, by SDI, regional number of DALYs, ASDR, and EAPC changes for 1990 and 2021

Location	Number 1990	ASR 1990	Number 2021	ASR 2021	EAPC_95%CI
Global	4909874.46 (1936762.4-7896305.18)	234.01 (93.58-380.47)	5662904.86 (2246472.2-9077004.16)	120.94 (47.2-197.93)	-2.5 (-2.61 to -2.39)
High SDI	347210.32 (138889.35-561704.1)	57.01 (22.64-93.47)	359565.29 (144011.77-585561.32)	31.79 (12.45-52.6)	-2 (-2.06 to -1.95)
High-middle SDI	1260020.28 (494751.88-2070646.15)	244.9 (97.09-401.29)	1096295.35 (438799.79-1760622.94)	101.96 (40.03-169.04)	-3.45 (-3.65 to -3.25)
Middle SDI	2073847.19 (834813.58-3331414.46)	415.46 (166.61-678.28)	2132844.46 (869137.14-3478022.09)	157.02 (61.04-259.51)	-3.64 (-3.8 to -3.48)
Low-middle SDI	981139.68 (388553.83-1631098.29)	319.31 (127.03-531.38)	1678458.37 (651581.88-2742202.54)	230.16 (88.18-381.63)	-1.08 (-1.16 to -0.99)
Low SDI	245192.43 (96769.98-408844.69)	217.76 (86.39-368.94)	392852.57 (148021.95-647490.07)	158.9 (60.16-268.39)	-1.07 (-1.19 to -0.96)
Andean Latin America	3469.21 (1343.03-5817.69)	32.02 (12.1-54)	4589.73 (1687.95-7824.69)	14.35 (5.2-25.2)	-2.71 (-2.81 to -2.62)
Australasia	7096.46 (2799.63-11642.18)	54.48 (20.15-93.99)	6214.76 (2420.48-10235.88)	21.39 (8.07-37.64)	-3.2 (-3.39 to -3)
Caribbean	5763.33 (2291.81-9531.08)	41.36 (16.15-69.23)	10920.27 (4319.75-18456.52)	36.54 (13.8-61.98)	-0.72 (-0.96 to -0.47)
Central Asia	29050.52 (11680.23-46843.23)	114.5 (45.3-185.76)	27931.38 (10975.84-45508.06)	65.16 (25.25-107.44)	-2.19 (-2.43 to -1.95)
Central Europe	75327.92 (30037.41-120548.01)	93.29 (37.01-151.05)	57254.04 (22577.4-94205.83)	48.08 (18.82-79.19)	-1.95 (-2.06 to -1.84)
Central Latin America	38395.05 (15694.46-61738.87)	92.11 (36.67-148.83)	57062.25 (21982.49-93628.23)	42.68 (16.65-70.88)	-2.81 (-2.96 to -2.67)
Central Sub-Saharan Africa	10655.12 (4123.82-19766.25)	90 (31.75-171.84)	18263.81 (7229.77-34042.87)	62.04 (22.28-121.83)	-1.44 (-1.53 to -1.35)
East Asia	2641627.88 (1038968.71-4308539.62)	688.37 (272.24-1136.16)	2248318.53 (903623.59-3666744.15)	204.26 (78.89-345.04)	-4.5 (-4.69 to -4.32)
Eastern Europe	147303.53 (60199.58-233961.99)	97.89 (38.73-160.25)	57027.6 (22163.54-93926.28)	30.37 (11.52-52)	-4.57 (-4.9 to -4.24)
Eastern Sub-Saharan Africa	32829.74 (13207.88-55238.18)	80.45 (31.9-138.06)	44459.5 (17661.6-76700.89)	45.84 (17.92-79.74)	-2.13 (-2.23 to -2.03)
High-income Asia Pacific	39162.99 (15409.87-62955.04)	37.09 (13.95-62.36)	40581.48 (15854.09-68493.55)	15 (5.55-26.37)	-3.07 (-3.14 to -3)

High-income North America	105775.7 (41905.31-174650.52)	55.58 (21.17-93.4)	136436.81 (52926.14-224221.35)	38.58 (14.61-66.07)	-1.29 (-1.42 to -1.16)
North Africa and Middle East	104419.96 (40548.05-172008.68)	121.13 (47.53-202.79)	199762.98 (78171.03-329071.82)	85.8 (33.02-143.81)	-1.14 (-1.24 to -1.05)
Oceania	7588.67 (2745.44-13316.59)	531.14 (190.91-937.19)	16580.6 (6480.7-26784.94)	455.72 (174.23-776.5)	-0.65 (-0.72 to -0.58)
South Asia	1115260.94 (453699.35-1849152.18)	396.68 (159.18-662.76)	2006642.47 (761516.79-3303092.77)	268.57 (100.8-450.49)	-1.32 (-1.42 to -1.21)
Southeast Asia	255428.89 (102989.93-417111.23)	198.84 (79.38-328.1)	427798.28 (164692.15-698087.29)	128.03 (48.92-210.34)	-1.63 (-1.73 to -1.52)
Southern Latin America	20920.98 (8242.9-34078.42)	83.83 (32.73-136.75)	24540.21 (9770.72-39759.45)	50.72 (19.27-84.04)	-1.59 (-1.83 to -1.35)
Southern Sub-Saharan Africa	22025.42 (8749.56-35607.92)	149.51 (58.42-247.15)	34230.73 (13587.35-54826.22)	108.6 (41.96-180.07)	-1.17 (-1.45 to -0.89)
Tropical Latin America	68082.58 (28071.7-108247.33)	145.8 (58.56-236.36)	71521.74 (28143.75-119572.88)	50.83 (19.4-88.19)	-4.04 (-4.35 to -3.73)
Western Europe	153646.92 (59755.43-251099.52)	49.1 (19.16-80.62)	131535.6 (52351.3-216556.47)	26.29 (10.21-44.02)	-1.93 (-1.98 to -1.88)
Western Sub-Saharan Africa	26042.63 (10350.67-44036.77)	53.72 (21.07-91.31)	41232.09 (15941.42-70329.9)	36.64 (13.98-62.07)	-1.25 (-1.29 to -1.2)

SDI - socio-demographic index, DALYs - disability-adjusted life years, ASDR - age-standardized disability-adjusted life year rates, EAPC - estimated annual percentage change.

Supplementary Table S3. Global, by SDI, regional YLLs, AS-YLLs, and EAPC changes for 1990 and 2021

Location	Number 1990	ASR 1990	Number 2021	ASR 2021	EAPC_95%CI
Global	572242.86 (222650.51-922524.29)	25.64 (9.93-42.55)	1056921.35 (404197.03-1718828.66)	22.33 (8.6-37.68)	-0.53 (-0.59 to -0.46)
High SDI	82105.49 (31687.2-135028.19)	13.9 (5.28-23.56)	106381.6 (40498.81-176533.31)	10.12 (3.82-17.09)	-1.06 (-1.15 to -0.98)
High-middle SDI	137089.56 (54018.97-221000.19)	25.02 (9.69-41.51)	244356.46 (94834.27-399786.61)	22.97 (8.9-38.87)	-0.36 (-0.4 to -0.31)
Middle SDI	193910.23 (76434.81-314305.12)	33.64 (13.2-55.62)	394505.95 (152255.38-637190.49)	27.13 (10.46-45.69)	-0.79 (-0.84 to -0.75)
Low-middle SDI	125281.52 (48588.42-207798.81)	37.12 (14.45-62.07)	246332.08 (95185.67-407643.23)	31.26 (12.09-53.36)	-0.62 (-0.76 to -0.49)
Low SDI	33488.18 (12684.96-55621.98)	26.1 (9.96-44.07)	64800.07 (24057.42-109261.93)	22.12 (8.29-38.17)	-0.65 (-0.83 to -0.47)
Andean Latin America	614.95 (223.8-1054.65)	5.18 (1.83-9.13)	1149.82 (401.09-1951.38)	3.47 (1.2-6.21)	-1.68 (-1.87 to -1.49)
Australasia	1103.89 (427.53-1848.46)	8.7 (3.17-15.5)	1150.52 (444.05-1937.81)	4.22 (1.5-7.67)	-2.41 (-2.55 to -2.28)
Caribbean	960.85 (360.42-1632.68)	6.67 (2.5-11.61)	1785.69 (670.05-3016.32)	6 (2.25-10.4)	-0.63 (-0.83 to -0.43)
Central Asia	3910.48 (1462.1-6356.06)	14.79 (5.54-25.07)	6092.22 (2212.07-10023.8)	13.72 (5.11-23.27)	-0.13 (-0.18 to -0.08)

Central Europe	12719.81 (4847.12-21068.43)	15.9 (5.96-27.21)	14671.58 (5569.39-24721.54)	12.95 (4.84-22.12)	-0.62 (-0.66 to -0.58)
Central Latin America	5628.03 (2096.3-9296.33)	12 (4.38-20.32)	10615.46 (3795.27-17643.77)	7.74 (2.81-13.26)	-1.6 (-1.69 to -1.51)
Central Sub-Saharan Africa	1907.15 (690.01-3399.53)	13.63 (4.87-24.94)	4234.32 (1508.35-7341.78)	12.15 (4.28-22.02)	-0.54 (-0.6 to -0.47)
East Asia	209635.75 (84258.38-339016.67)	44.26 (17.39-74.07)	413919.2 (161648.27-681669.19)	35.52 (13.65-60.4)	-0.75 (-0.83 to -0.66)
Eastern Europe	23615.26 (9103.72-38886.84)	15.84 (6.09-27.02)	17352.23 (6395.28-29069.96)	9.58 (3.51-16.62)	-1.79 (-2.01 to -1.57)
Eastern Sub-Saharan Africa	6411.29 (2403.47-10923.14)	13.13 (4.78-22.89)	12105.23 (4451.75-20972.67)	10.4 (3.75-18.36)	-1 (-1.08 to -0.92)
High-income Asia Pacific	13858.52 (5154.33-23267.44)	12.73 (4.6-22.15)	18112.2 (6634.16-30484.33)	7.71 (2.74-13.81)	-1.68 (-1.84 to -1.51)
High-income North America	31106.22 (11954.49-51105.03)	16.81 (6.27-28.91)	39022.53 (14589.31-64594.51)	11.56 (4.27-20.32)	-1.27 (-1.42 to -1.12)
North Africa and Middle East	19412.13 (7491.76-31815.84)	20.22 (7.72-34.22)	55570.46 (22128.08-92103.04)	21.73 (8.31-36.66)	0.08 (0.01 to 0.16)
Oceania	543.49 (207.45-897.72)	33.68 (12.66-56.53)	1408.89 (550.53-2293.13)	33.33 (12.82-56.49)	-0.15 (-0.2 to -0.09)
South Asia	149035.97 (58096.26-249241.11)	47.77 (18.58-80.2)	306977.68 (118715.55-511596.14)	38.26 (14.62-66)	-0.83 (-1.02 to -0.64)
Southeast Asia	37388.67 (14580.15-60159.96)	25.65 (9.85-43.18)	82003.03 (31359.36-134370.73)	22.85 (8.69-38.75)	-0.52 (-0.58 to -0.46)
Southern Latin America	2172.09 (817.49-3487.6)	8.63 (3.11-14.94)	3069.68 (1115.97-5037.92)	6.5 (2.38-11.31)	-0.97 (-1.07 to -0.86)
Southern Sub-Saharan Africa	5911.15 (2198.83-9802.84)	37.57 (14.15-63.89)	9252.84 (3412.73-15563.93)	27.75 (10.37-48.06)	-1.03 (-1.09 to -0.98)
Tropical Latin America	7209.63 (2785.32-12217.47)	14.26 (5.4-24.21)	10782.23 (4067.21-18568.99)	7.63 (2.74-13.72)	-2.31 (-2.43 to -2.19)
Western Europe	32791.71 (12343.26-53837.54)	11.19 (4.28-18.91)	34382.86 (13246.31-57623.78)	7.73 (2.96-13.25)	-1.18 (-1.28 to -1.08)
Western Sub-Saharan Africa	6305.81 (2307.13-10541.05)	11.4 (4.16-19.93)	13262.68 (4751.21-22702.21)	10.14 (3.66-17.86)	-0.52 (-0.6 to -0.45)

SDI - socio-demographic index, YLLs - years of life lost, AS-YLLs - age-standardized years of life lost, EAPC - estimated annual percentage change.

Supplementary Table S4. Global, by SDI, regional YLDs, AS-YLD rates, and EAPC changes for 1990 and 2021

Location	Number 1990	ASR 1990	Number 2021	ASR 2021	EAPC_95%CI
Global	4337631.6 (1723025.55-7019744.11)	208.36 (83.19-339.94)	4605983.52 (1832410.28-7469197.36)	98.61 (38.45-162.17)	-2.83 (-2.96 to -2.7)
High SDI	265104.83 (105942.97-435940.63)	43.11 (17.22-70.54)	253183.69 (100476.18-414220.75)	21.68 (8.61-36)	-2.37 (-2.45 to -2.29)
High-middle SDI	1122930.72 (439667.69-1843931.02)	219.88 (87.17-361.49)	851938.89 (343079.13-1395105.01)	78.99 (30.97-133.07)	-4.01 (-4.25 to -3.78)
Middle SDI	1879936.96 (753853.38-3041119.12)	381.83 (152.67-626.44)	1738338.51 (710393.27-2849852.96)	129.88 (50.52-217.04)	-4.04 (-4.21 to -3.86)
Low-middle SDI	855858.16 (335675.81-1436437.64)	282.2 (111.64-473.6)	1432126.29 (562711.16-2359927.23)	198.9 (75.95-330.7)	-1.14 (-1.23 to -1.06)
Low SDI	211704.25 (83960.2-355534.34)	191.66 (75.8-327.11)	328052.5 (124286.38-544149.05)	136.78 (51.66-231.56)	-1.14 (-1.26 to -1.02)
Andean Latin America	2854.26 (1130.35-4750.97)	26.85 (10.16-45.58)	3439.91 (1273.92-5907.28)	10.88 (3.9-19.43)	-2.98 (-3.07 to -2.89)
Australasia	5992.57 (2362.87-9972.12)	45.78 (16.93-79.43)	5064.24 (1957.59-8512.29)	17.17 (6.44-30.49)	-3.38 (-3.64 to -3.12)
Caribbean	4802.48 (1948.37-7922.53)	34.69 (13.55-57.88)	9134.58 (3610.89-15518.16)	30.53 (11.49-52.13)	-0.73 (-0.99 to -0.47)
Central Asia	25140.04 (10104.95-40650.93)	99.71 (39.75-162.51)	21839.16 (8451.57-36054.95)	51.43 (19.79-85.72)	-2.58 (-2.86 to -2.3)
Central Europe	62608.11 (25359.8-101106.08)	77.4 (30.83-125.33)	42582.46 (16859.97-71293.75)	35.13 (13.73-58.12)	-2.33 (-2.47 to -2.2)
Central Latin America	32767.02 (13425.78-52834.96)	80.11 (31.99-129.45)	46446.79 (17661.33-75606.13)	34.94 (13.69-58.28)	-3.04 (-3.2 to -2.88)
Central Sub-Saharan Africa	8747.97 (3248.25-16768.95)	76.37 (26.29-149.87)	14029.49 (5481.55-27117.64)	49.89 (17.46-102.48)	-1.63 (-1.72 to -1.54)
East Asia	2431992.13 (949278.63-3973624.55)	644.1 (254.26-1067.63)	1834399.32 (742038.98-3033253.03)	168.74 (65-288.55)	-4.96 (-5.17 to -4.75)
Eastern Europe	123688.27 (50950.69-197387.45)	82.05 (32.51-134.53)	39675.37 (15295.8-66357.92)	20.79 (7.89-36.02)	-5.35 (-5.74 to -4.96)
Eastern Sub-Saharan Africa	26418.45 (10623.58-44901.6)	67.31 (26.59-116.12)	32354.27 (12638.68-55903.11)	35.43 (13.89-62.26)	-2.39 (-2.5 to -2.29)
High-income Asia Pacific	25304.47 (9851.01-41293.19)	24.36 (9.24-40.91)	22469.29 (8681.63-38477.78)	7.29 (2.72-13.01)	-4.18 (-4.35 to -4)
High-income North America	74669.48 (29629.84-123171.63)	38.78 (14.88-64.62)	97414.28 (37812.67-160479.82)	27.02 (10.29-46.35)	-1.29 (-1.42 to -1.16)
North Africa and Middle East	85007.84 (33675.85-141655.07)	100.91 (39.47-171.15)	144192.51 (57045.1-237202.62)	64.07 (24.5-108.71)	-1.47 (-1.6 to -1.34)
Oceania	7045.18 (2532.78-12448.2)	497.46 (177.11-885.5)	15171.71 (5890.07-24797.53)	422.39 (160.9-725.3)	-0.69 (-0.76 to -0.62)
South Asia	966224.97 (390682.75-1620476.85)	348.91 (138.96-587.12)	1699664.79 (654080.22-2772078.48)	230.31 (85.61-385.44)	-1.39 (-1.49 to -1.29)
Southeast Asia	218040.22 (87725.51-354311.14)	173.19 (68.63-287.26)	345795.25 (134466.75-568713.81)	105.17 (40-173.05)	-1.82 (-1.94 to -1.71)

Southern Latin America	18748.89 (7430.01-30361.87)	75.2 (29.56-122.88)	21470.53 (8497.43-34766.56)	44.22 (16.93-73.71)	-1.66 (-1.92 to -1.4)
Southern Sub-Saharan Africa	16114.27 (6581.2-26042.16)	111.93 (43.93-188.86)	24977.88 (10044.52-40207.97)	80.85 (31.24-134.61)	-1.21 (-1.57 to -0.85)
Tropical Latin America	60872.95 (25291.39-97021.16)	131.53 (53.14-213.85)	60739.52 (23923.29-101496.42)	43.2 (16.45-75.25)	-4.27 (-4.6 to -3.93)
Western Europe	120855.21 (47569.02-197721.56)	37.92 (14.87-62.21)	97152.74 (38685.03-159625.72)	18.56 (7.26-31.05)	-2.2 (-2.27 to -2.13)
Western Sub-Saharan Africa	19736.82 (8006.11-34477.36)	42.32 (16.7-72.67)	27969.41 (10979.97-47414.33)	26.49 (10.14-45.28)	-1.48 (-1.53 to -1.43)

SDI - socio-demographic index, YLDs - years of life disabled, AS-YLDs - age-standardized years of life disabled, EAPC - estimated annual percentage change.

Supplementary Table S5. Global, by SDI, Region, 204 countries, 2021 number of deaths, DALYs, ASMR, ASDR, Percentage change in number of deaths, Percentage change in number of DALYs

Location	Number of Deaths 2021	Number of DALYs 2021	ASMR 2021	ASDR 2021	Percentage Change in Deaths	Percentage Change in DALYs
Global	266124.7 (106054.27-434215.02)	5662904.86 (2246472.2-9077004.16)	5.87 (2.29-9.72)	120.94 (47.2-197.93)	22.55%	15.34%
High SDI	14985.48 (5966.28-24623.12)	359565.29 (144011.77-585561.32)	1.16 (0.46-1.94)	31.79 (12.45-52.6)	6.83%	3.56%
High-middle SDI	55393.03 (22274.34-92140.47)	1096295.35 (438799.79-1760622.94)	5.23 (2.05-8.86)	101.96 (40.03-169.04)	-7.69%	-12.99%
Low SDI	16190.95 (6161.47-26927.79)	392852.57 (148021.95-647490.07)	7.98 (3.02-13.49)	158.9 (60.16-268.39)	69.72%	60.22%
Low-middle SDI	74546.14 (29434.4-123115.06)	1678458.37 (651581.88-2742202.54)	11.57 (4.44-19.25)	230.16 (88.18-381.63)	87.04%	71.07%
Middle SDI	104890.54 (42778.94-172646.77)	2132844.46 (869137.14-3478022.09)	8.55 (3.32-14.35)	157.02 (61.04-259.51)	12.04%	2.84%
Afghanistan	257.49 (89.25-463.02)	7656.75 (2677.26-13608.74)	6.14 (1.99-11.81)	142.86 (47.71-267.71)	40.94%	56.45%
Albania	50.34 (18.99-86.48)	1161.79 (436.69-1944.51)	2.23 (0.78-4.16)	49.77 (18.1-88.75)	-8.61%	-1.84%
Algeria	486.1 (180.47-857.31)	14757.96 (5762.55-24770.06)	3.53 (1.25-6.59)	82.12 (30.27-144.14)	202.19%	189.72%
American Samoa	1.74 (0.68-2.87)	40.56 (15.99-66.85)	8.69 (3.24-15.26)	171.38 (64.04-297.57)	47.46%	36.15%
Andean Latin America	215 (80.12-369.05)	4589.73 (1687.95-7824.69)	0.7 (0.25-1.25)	14.35 (5.2-25.2)	38.71%	32.30%
Andorra	1 (0.38-1.78)	24.78 (9.53-42.93)	1.09 (0.36-2.2)	28.97 (10.28-55.26)	29.87%	24.02%
Angola	68.87 (24.57-119.2)	2532.67 (917.71-4299.49)	1.43 (0.48-2.74)	37.86 (13.04-69.61)	70.98%	84.97%
Antigua and Barbuda	0.27 (0.1-0.45)	6.89 (2.72-11.62)	0.52 (0.18-0.96)	12.22 (4.25-22.19)	125.00%	146.95%

Argentina	775.06 (296.2-1270.06)	16611.79 (6526.9-26905.32)	2.44 (0.9-4.19)	54.17 (20.26-91.54)	29.97%	18.86%
Armenia	57.82 (22.32-94.61)	1286.69 (511.5-2053.64)	2.42 (0.92-4.03)	54.22 (20.68-89.52)	-37.51%	-40.90%
Australasia	287.77 (111.23-493.22)	6214.76 (2420.48-10235.88)	0.9 (0.33-1.62)	21.39 (8.07-37.64)	-3.58%	-12.42%
Australia	234.07 (91.97-403.13)	5000.17 (1990.71-8291.52)	0.86 (0.31-1.6)	20.37 (7.57-36.87)	-4.80%	-14.39%
Austria	114.5 (45.35-193.12)	2986.21 (1162.75-4931.42)	1.07 (0.4-1.89)	31.73 (11.81-54.96)	32.10%	33.22%
Azerbaijan	111.51 (43-200.96)	3270.61 (1256.87-5829.17)	2.56 (0.95-4.87)	63.07 (23.29-114.73)	3.65%	19.03%
Bahamas	1.34 (0.51-2.32)	34.82 (13.15-59.64)	0.69 (0.23-1.27)	16.03 (5.49-29.13)	191.30%	180.35%
Bahrain	10.33 (3.9-17.24)	336.38 (129.64-558.34)	4.5 (1.61-7.96)	87.45 (31.71-153.53)	140.23%	176.97%
Bangladesh	6335.83 (2528.46-10836.3)	155118.57 (63035.26-261393.72)	9.66 (3.67-17.23)	213.89 (83.66-371.77)	69.89%	67.92%
Barbados	0.75 (0.26-1.26)	18.57 (6.35-31.34)	0.26 (0.09-0.47)	6.6 (2.27-11.94)	36.36%	44.74%
Belarus	54.73 (20.47-90.49)	2006.88 (781.91-3240.81)	0.61 (0.22-1.08)	23.61 (8.71-40.34)	-83.88%	-73.13%
Belgium	163.53 (64.84-285.69)	3683.3 (1455.03-6386.71)	1.15 (0.42-2.11)	30.28 (11.03-54.54)	-30.25%	-29.90%
Belize	1.95 (0.75-3.28)	49.38 (19.03-84.39)	1.34 (0.49-2.41)	30.25 (11.05-53.54)	267.92%	283.68%
Benin	24.49 (8.46-43.65)	862.82 (316.64-1510.04)	1.05 (0.35-2.05)	28.57 (9.82-54.23)	4.48%	28.11%
Bermuda	0.57 (0.21-1.01)	11.92 (4.54-20.43)	0.67 (0.23-1.26)	15.42 (5.41-28.25)	90.00%	73.51%
Bhutan	24.44 (8.38-42.67)	516.98 (182.71-876.35)	8.35 (2.82-15.66)	163.5 (55.91-297.3)	105.55%	60.72%
Bolivia (Plurinational State of)	63.97 (22.03-115.72)	1418.18 (492.35-2589.56)	1.67 (0.53-3.23)	31.38 (10.19-59.55)	62.07%	52.11%
Bosnia and Herzegovina	79.95 (29.4-136.84)	1930.85 (720.92-3266.52)	2.25 (0.81-4.01)	56.03 (20.1-98.31)	7.13%	0.60%
Botswana	26.77 (9.55-45.67)	828.29 (311.69-1365.06)	4.3 (1.44-8.15)	106.63 (37.43-192.72)	23.25%	37.90%
Brazil	3113.2 (1205.46-5164.53)	70227.46 (27655.48-117428.02)	2.33 (0.88-4.08)	51.05 (19.5-88.75)	5.90%	4.05%
Brunei Darussalam	2.6 (1-4.34)	67.58 (26.58-112.02)	2.15 (0.76-4.03)	41.51 (14.71-75.99)	-1.14%	12.80%
Bulgaria	135.75 (50.04-229.25)	3475.31 (1307.33-5725.02)	1.73 (0.62-3.06)	46.85 (16.95-81.79)	-44.89%	-45.18%
Burkina Faso	64.69 (24.2-116.02)	2279.34 (852.41-3936.79)	1.59 (0.55-3.08)	44.09 (15.6-81.83)	40.17%	56.66%
Burundi	29.13 (11.16-50.25)	1099.75 (428.66-1876.89)	1.26 (0.44-2.43)	36.13 (13-67.09)	-23.78%	-6.12%
Cabo Verde	2.53 (0.94-4.41)	70.74 (26.08-119.23)	1.1 (0.36-2.17)	28.29 (9.57-53.73)	-35.46%	-18.61%

Cambodia	299.89 (110.71-508.29)	7574.21 (2980.1-12547.84)	6.43 (2.25-11.56)	127.73 (46.64-224.44)	158.99%	147.16%
Cameroon	73.98 (26.52-130.32)	2582.58 (960.12-4470.91)	1.36 (0.46-2.57)	35.54 (12.33-65.04)	33.42%	60.95%
Canada	408.62 (159.21-697.9)	9785.15 (3852.5-16417.7)	0.95 (0.34-1.67)	25.01 (9-43.32)	4.02%	1.66%
Caribbean	507.74 (201.08-857.91)	10920.27 (4319.75-18456.52)	1.68 (0.63-2.87)	36.54 (13.8-61.98)	101.16%	89.48%
Central African Republic	28.77 (10.28-55.72)	989 (368.23-1840.8)	3.2 (1.03-6.88)	79.5 (26.82-160.8)	30.42%	42.03%
Central Asia	1027.67 (401.14-1684.33)	27931.38 (10975.84-45508.06)	2.75 (1.07-4.58)	65.16 (25.25-107.44)	-13.77%	-3.85%
Central Europe	2284.87 (911.21-3816.8)	57254.04 (22577.4-94205.83)	1.78 (0.69-2.94)	48.08 (18.82-79.19)	-25.60%	-23.99%
Central Latin America	2831.26 (1073.58-4626.91)	57062.25 (21982.49-93628.23)	2.2 (0.87-3.68)	42.68 (16.65-70.88)	56.78%	48.62%
Central Sub-Saharan Africa	566.19 (216.57-1120.96)	18263.81 (7229.77-34042.87)	2.61 (0.9-5.47)	62.04 (22.28-121.83)	62.04%	71.41%
Chad	46.8 (16.9-87.57)	1627.05 (626.66-2899.4)	1.81 (0.61-3.51)	48.43 (16.95-90.44)	38.95%	62.78%
Chile	358.84 (139.2-604.17)	6117.22 (2383.4-10040.05)	2.45 (0.92-4.25)	42.38 (15.9-72.99)	44.90%	18.97%
China	117387.96 (47776.16-195915.33)	2182250.92 (876774.91-3556235.66)	12.25 (4.7-21.09)	206.32 (79.57-349.89)	-5.19%	-16.04%
Colombia	802.28 (301.87-1380.71)	14255.16 (5363.32-24205.21)	2.52 (0.92-4.49)	46.02 (16.82-81.1)	101.85%	58.04%
Comoros	6.34 (2.37-11.48)	184.11 (72.51-323.4)	2.9 (0.99-5.54)	70.28 (25.12-131.14)	44.75%	48.22%
Congo	27.54 (9.53-48.61)	912.11 (335-1590.19)	2.48 (0.81-4.85)	61.41 (20.82-116.31)	30.58%	49.08%
Cook Islands	0.54 (0.21-0.9)	11.56 (4.6-18.95)	3.99 (1.44-7.29)	83.31 (30.41-147.53)	5.88%	-1.95%
Costa Rica	47.84 (18.38-79.99)	910.31 (350.64-1490.9)	1.51 (0.55-2.67)	29.52 (10.82-51.43)	63.67%	52.17%
Croatia	164.89 (64.64-270.78)	3261.84 (1325.15-5290.41)	2.95 (1.11-4.99)	63.56 (24.17-107.92)	64.18%	23.95%
Cuba	300.5 (117.99-509.85)	6198.42 (2464.7-10546.63)	2.62 (0.96-4.51)	56.63 (20.9-97.04)	122.38%	105.41%
Cyprus	20.1 (7.69-34.28)	441.44 (173.19-724.11)	2.18 (0.74-4.11)	42.51 (15.21-76.69)	35.63%	35.54%
Czechia	213.7 (77.37-357.16)	5085.47 (1896.15-8386.19)	1.68 (0.61-2.97)	43.6 (15.76-76.81)	13.55%	5.22%
C 么 te d'Ivoire	110.25 (41.22-188.12)	3921.24 (1500.84-6575.23)	2.39 (0.81-4.51)	61.92 (21.83-112.37)	85.17%	100.92%
Democratic People's Republic of Korea	2754.73 (1115.05-5119.61)	54699.6 (21909.03-98436.16)	17.95 (6.44-35.91)	324.32 (120.36-619.58)	67.18%	53.50%
Democratic Republic of the Congo	426.07 (155.19-892.3)	13335.23 (5093.59-26272.1)	2.95 (0.94-6.65)	68.71 (23.27-143.52)	69.84%	76.23%
Denmark	117.69 (46.3-201.65)	2387.72 (954.73-4023.16)	1.64 (0.6-3.01)	37.38 (13.88-67.61)	-31.57%	-42.31%

Djibouti	6.52 (2.26-12.2)	233.69 (87.33-413.72)	2.79 (0.88-5.5)	70.42 (24.39-130.34)	207.55%	236.92%
Dominica	0.54 (0.2-0.93)	12 (4.67-20.14)	1.26 (0.41-2.41)	26.46 (8.91-49.89)	20.00%	28.48%
Dominican Republic	52.96 (19.16-97.58)	1199.91 (445.06-2135.42)	1 (0.33-2.05)	22.01 (7.54-42.47)	156.22%	154.86%
East Asia	120731.24 (49030.26-200484.49)	2248318.53 (903623.59-3666744.15)	12.06 (4.64-20.66)	204.26 (78.89-345.04)	-3.97%	-14.89%
Eastern Europe	1935.57 (757.13-3190.37)	57027.6 (22163.54-93926.28)	0.98 (0.37-1.7)	30.37 (11.52-52)	-68.19%	-61.29%
Eastern Sub-Saharan Africa	1325.44 (518.6-2314.93)	44459.5 (17661.6-76700.89)	1.75 (0.69-3.09)	45.84 (17.92-79.74)	21.96%	35.42%
Ecuador	77.96 (30.1-135.51)	1482.15 (559.65-2523.21)	1.03 (0.37-1.86)	17.7 (6.34-31.88)	36.13%	33.26%
Egypt	789.72 (304.08-1329.05)	26390.58 (10358.98-44029.01)	3.38 (1.21-6.13)	81.78 (30.4-143.56)	5.02%	23.48%
El Salvador	42.07 (15.41-73.18)	860.84 (311.55-1458.63)	1.11 (0.38-2.05)	24.18 (8.39-43.49)	105.62%	91.06%
Equatorial Guinea	5.91 (2.05-10.85)	204.97 (73.87-367.96)	2.91 (0.9-5.87)	74.2 (24.44-140.23)	4.79%	25.52%
Eritrea	27.56 (10.5-50.23)	966.08 (372.43-1710.66)	2.29 (0.8-4.57)	59.17 (20.73-114.71)	61.64%	58.36%
Estonia	8.9 (3.32-14.83)	213.66 (79.51-356.08)	0.52 (0.18-0.94)	14.54 (5.21-25.8)	-27.41%	-37.54%
Eswatini	10.29 (3.86-18.35)	322.55 (122.4-561.42)	3.92 (1.33-7.69)	100.62 (35.24-190.2)	20.49%	35.71%
Ethiopia	220.31 (89.53-387.96)	7892.23 (3204.62-13865.25)	1.05 (0.38-1.95)	30.33 (11.32-55.49)	-10.48%	-4.34%
Fiji	16.32 (6.05-28.42)	406.39 (152.58-689.02)	5.95 (2.1-11.02)	114.67 (40.95-207.88)	23.82%	19.59%
Finland	29.51 (10.62-50.21)	780.76 (302.29-1315.29)	0.38 (0.13-0.7)	12.33 (4.36-22.51)	-2.16%	-14.43%
France	468.18 (172.01-803)	10484.38 (4033.22-17490.61)	0.49 (0.17-0.89)	14.39 (5.18-25.5)	-20.80%	-14.89%
Gabon	9.03 (3.15-16.32)	289.83 (104.48-505.62)	2.03 (0.65-4.07)	51.28 (17.4-97.22)	-4.85%	17.19%
Gambia	15.47 (6.14-28.88)	462.93 (185-826.68)	3.57 (1.28-7.09)	86.26 (32.23-164.07)	106.27%	109.80%
Georgia	60.95 (23.54-100.25)	1582.21 (614.35-2546.19)	1.72 (0.65-2.95)	47.66 (18.07-79.96)	28.18%	17.68%
Germany	1062.76 (404.51-1774.91)	26744.15 (9899.6-44455.87)	0.95 (0.34-1.73)	27.82 (10.15-49.33)	13.15%	8.56%
Ghana	40.24 (14.01-70.86)	1853.89 (683.87-3171.26)	0.52 (0.17-1)	17.98 (6.26-33.16)	112.91%	124.01%
Greece	249.29 (93.98-414.45)	5475.5 (2079.77-8840.97)	1.41 (0.52-2.45)	39.91 (14.89-68.6)	73.13%	35.64%
Greenland	1.41 (0.53-2.44)	38.89 (14.79-65.22)	4.23 (1.47-7.88)	100.29 (35.52-182.06)	-10.76%	-4.75%
Grenada	0.52 (0.21-0.87)	13.14 (5.26-21.93)	0.92 (0.32-1.7)	21.34 (7.66-39.06)	40.54%	66.75%

Guam	2.51 (1.01-4.11)	68.2 (26.91-109.08)	2.07 (0.77-3.7)	58.39 (22.04-101.55)	34.22%	39.98%
Guatemala	65.83 (24.43-112.74)	1507.71 (553.8-2529.99)	1.3 (0.46-2.33)	26.35 (9.39-46.6)	151.45%	126.98%
Guinea	62.43 (22.97-111.54)	2012.71 (718.08-3362.94)	2.38 (0.82-4.5)	62.9 (22.47-113.94)	12.89%	31.79%
Guinea-Bissau	6.67 (2.5-11.99)	241.12 (91.3-428.41)	2.14 (0.76-4.2)	55.63 (19.91-104.84)	40.42%	62.76%
Guyana	3.31 (1.22-5.83)	90.43 (33.37-158.63)	1.09 (0.37-2.01)	25.93 (9.12-47.33)	63.86%	73.80%
Haiti	51.42 (16.76-100.95)	1347.3 (469.31-2641.11)	1.72 (0.5-3.58)	36.16 (11.08-74.61)	36.28%	38.60%
High-income Asia Pacific	1744.15 (658.1-2969.58)	40581.48 (15854.09-68493.55)	0.49 (0.18-0.87)	15 (5.55-26.37)	14.40%	3.62%
High-income North America	5043.88 (1966.67-8330.45)	136436.81 (52926.14-224221.35)	1.32 (0.5-2.29)	38.58 (14.61-66.07)	38.77%	28.99%
Honduras	170.86 (62.68-294.03)	3780.72 (1391.81-6509.31)	6.35 (2.18-11.74)	119.13 (41.84-217.84)	246.15%	221.22%
Hungary	291.06 (109.57-492.33)	7436.45 (2758.33-12351.74)	2.7 (0.98-4.69)	75.71 (27.79-130.37)	-9.06%	-5.55%
Iceland	2.31 (0.88-3.92)	60.31 (22.7-100.43)	0.65 (0.23-1.17)	19.37 (7-34.83)	0.43%	-5.85%
India	75614.28 (28341.18-124842.16)	1669829.85 (619318.64-2735802.85)	14.16 (5.22-24.07)	276.64 (102.78-470.11)	106.40%	84.65%
Indonesia	6775.14 (2718.11-11353.03)	178956.03 (70409.56-294367.44)	7.27 (2.83-12.5)	152.17 (59.09-258.37)	126.58%	120.82%
Iran (Islamic Republic of)	519.45 (201.35-882.25)	15927.29 (6109.98-26331.65)	1.41 (0.52-2.48)	37.14 (13.94-64.27)	172.29%	160.10%
Iraq	191.11 (75.44-328.82)	6937.48 (2809.5-11391.04)	2 (0.74-3.68)	56.1 (21.56-96.8)	102.04%	135.28%
Ireland	67.65 (25.29-114.81)	1406.48 (529.17-2311.62)	1.44 (0.51-2.6)	32.01 (11.57-56.72)	-46.65%	-45.42%
Israel	55.37 (21.1-93.23)	1476.49 (574.64-2430.14)	0.76 (0.27-1.38)	22.36 (7.98-39.98)	17.93%	29.28%
Italy	971.34 (379.81-1646.27)	18632.42 (7379.62-31266.71)	0.92 (0.34-1.66)	22.47 (8.33-39.71)	-6.53%	-21.21%
Jamaica	19.47 (7.14-34.49)	429.11 (155.49-727.96)	1.07 (0.38-2.02)	24.6 (8.76-44.98)	68.43%	63.09%
Japan	1166.03 (424.09-2006.41)	25700.99 (9328.59-43805.09)	0.39 (0.13-0.72)	11.72 (4.05-21.34)	7.33%	-9.60%
Jordan	46 (18-77.01)	1940.6 (776.37-3151.8)	1.56 (0.56-2.81)	48.1 (18.24-82.27)	121.90%	194.73%
Kazakhstan	463.95 (176.44-761.34)	11689.52 (4439.19-18948.23)	5.56 (2.06-9.64)	122.65 (45.61-210.73)	14.03%	19.29%
Kenya	195.93 (70.5-430.08)	5986.4 (2182.28-11758.17)	2.03 (0.68-4.75)	48.02 (16.91-100.7)	90.13%	103.16%
Kiribati	5.49 (2.23-9.14)	146.41 (61.23-244.43)	21.72 (8.13-39.25)	424.09 (163.63-745.01)	49.59%	52.69%
Kuwait	4.4 (1.76-7.23)	439.4 (176.02-705.79)	0.38 (0.14-0.68)	23.96 (8.95-41.58)	104.65%	264.38%

Kyrgyzstan	91.38 (37.79-152.69)	2414.51 (992.46-3997.37)	4.45 (1.77-7.6)	98.32 (39.24-166.08)	-55.85%	-48.41%
Lao People's Democratic Republic	174.48 (68.35-307.21)	4347.12 (1686.08-7641.32)	9.49 (3.53-17.61)	191.8 (72.84-345.51)	39.46%	35.74%
Latvia	16.75 (6.32-27.81)	498.57 (189.76-820.03)	0.71 (0.26-1.26)	24.02 (8.74-41.78)	-48.37%	-42.71%
Lebanon	114.41 (44.34-191.11)	2668.69 (1059.77-4348.06)	3.11 (1.12-5.51)	76.87 (28.27-132.13)	161.33%	133.24%
Lesotho	47.09 (17.1-82.3)	1366.54 (508.65-2383.55)	9.29 (3.19-17.83)	230.89 (82.44-427.16)	78.37%	90.62%
Liberia	13.62 (4.88-23.73)	489.39 (181.91-839.83)	1.48 (0.5-2.82)	38.95 (13.64-71.75)	15.13%	45.53%
Libya	81.51 (32.53-142.73)	2686.79 (1082.27-4505.48)	3.35 (1.2-6.27)	91.05 (34.58-161.97)	182.73%	215.64%
Lithuania	26.07 (10.02-44.27)	684.56 (263.85-1139.19)	0.75 (0.27-1.36)	22.83 (8.2-40.55)	-64.48%	-60.97%
Luxembourg	6.74 (2.52-11.34)	158.32 (59.93-269.74)	1.05 (0.37-1.92)	27.07 (9.76-48.83)	5.64%	0.55%
Madagascar	161.09 (53-300.5)	5200.76 (1754.43-9423.22)	3.75 (1.24-7.36)	85.08 (28.11-162.84)	-6.99%	13.28%
Malawi	67.75 (25.57-121.04)	2391.36 (911.67-4213.75)	1.97 (0.68-3.77)	54.8 (19.45-100.83)	69.21%	83.20%
Malaysia	541.9 (207.48-881.69)	13781.27 (5301.65-22415.19)	4.18 (1.51-7.52)	92.94 (34.04-162.61)	100.85%	108.84%
Maldives	9.14 (3.52-14.98)	219.65 (87.55-356.98)	6.44 (2.36-11.19)	128.18 (48-218.36)	69.26%	43.56%
Mali	192.03 (69.09-335.11)	5825.88 (2111.26-10061.7)	4.92 (1.67-9.23)	118.52 (41.04-217.25)	123.71%	130.42%
Malta	3.16 (1.17-5.42)	82.18 (30.1-141.66)	0.53 (0.19-1)	16.29 (5.69-29.93)	-10.99%	-11.36%
Marshall Islands	1.3 (0.52-2.27)	37.08 (14.59-64.26)	11.14 (4.03-20.49)	221.22 (80.15-403.32)	22.64%	37.18%
Mauritania	24.44 (9.07-42.34)	766.71 (295.42-1290.06)	2.48 (0.86-4.6)	64.98 (23.52-115.76)	-2.94%	13.11%
Mauritius	24.6 (9.81-39.91)	590.24 (232.7-970.57)	2.75 (1.05-4.71)	62.18 (23.81-105.93)	45.22%	44.74%
Mexico	1261.57 (513.66-2080.37)	26012.87 (10126.97-42507.06)	2.07 (0.8-3.54)	39.46 (15.16-68.09)	12.44%	14.80%
Micronesia (Federated States of)	3.93 (1.48-6.53)	104.4 (39.13-174.82)	14.52 (5.34-26.16)	291.89 (107.43-520.62)	-21.71%	-12.36%
Monaco	0.4 (0.15-0.7)	9.66 (3.71-16.92)	0.65 (0.22-1.27)	19.41 (6.88-36.24)	-6.98%	-8.61%
Mongolia	24.78 (8.91-41.73)	705.99 (261.9-1173)	2.85 (0.99-5.05)	62.1 (22.18-106.82)	-7.26%	8.02%
Montenegro	4.67 (1.78-7.76)	150.25 (56.21-249.21)	0.97 (0.35-1.68)	29.27 (10.7-50.25)	60.48%	53.50%
Morocco	500.02 (196.77-860.08)	13641.59 (5561-23273.8)	3.17 (1.1-5.88)	75.57 (27.43-135.12)	122.70%	124.51%
Mozambique	86.59 (32.1-149.88)	3041.57 (1116.11-5161.83)	1.78 (0.59-3.48)	47.35 (15.86-88.32)	61.76%	76.02%

Myanmar	3085.59 (1154.93-5270.34)	65497.73 (24807.96-110491.94)	14.74 (5.27-26.29)	272.26 (98.77-481.07)	24.73%	11.31%
Namibia	34.47 (12.9-60.39)	977.82 (371.83-1660.42)	5.6 (1.95-10.38)	132.63 (47.51-242.21)	38.99%	42.39%
Nauru	0.35 (0.13-0.63)	9.37 (3.53-16.31)	16.66 (5.69-34.67)	329.53 (116.24-644.63)	-14.63%	-15.74%
Nepal	2030.27 (802.75-3355.16)	44142.45 (17542.47-73898.43)	20 (7.61-34.79)	377.88 (144.39-655.49)	79.75%	63.91%
Netherlands	246.46 (94.07-415.99)	5558.44 (2121.19-9480.47)	1.18 (0.42-2.14)	30 (10.84-53.57)	-2.63%	-6.76%
New Zealand	53.71 (20.32-89.23)	1214.59 (462.02-1972.62)	1.1 (0.39-1.98)	26.61 (9.59-47.29)	2.13%	-3.27%
Nicaragua	49.92 (18.32-84.98)	1180.41 (442.81-1950.25)	2.26 (0.77-4.08)	47.15 (16.29-82.44)	187.23%	173.08%
Niger	58.31 (20.02-104.19)	2090.69 (757.3-3583.1)	1.66 (0.55-3.36)	44.94 (15.25-86.69)	79.03%	96.14%
Nigeria	261.06 (97.26-440.46)	10453.49 (3871.16-17563.09)	0.64 (0.23-1.14)	19.5 (6.95-34.24)	4.19%	31.40%
Niue	0.08 (0.03-0.13)	1.58 (0.62-2.66)	7.01 (2.54-12.77)	137.19 (49.62-246.85)	-46.67%	-42.12%
North Africa and Middle East	7325.96 (2889.15-12015.13)	199762.98 (78171.03-329071.82)	3.81 (1.45-6.51)	85.8 (33.02-143.81)	85.92%	91.31%
North Macedonia	60.7 (22.91-108.43)	1474.98 (560.85-2546.97)	4.25 (1.53-7.76)	88.71 (32.94-157.73)	13.78%	20.91%
Northern Mariana Islands	1.47 (0.58-2.4)	37.15 (14.37-59.46)	7.88 (2.89-13.97)	152.66 (55.78-267.73)	122.73%	87.25%
Norway	63.87 (23.99-110.8)	1381.51 (528.56-2349.87)	1.05 (0.37-1.92)	25.42 (9.19-45.65)	40.71%	14.80%
Oceania	668.65 (262.57-1089.5)	16580.6 (6480.7-26784.94)	23.29 (8.87-39.65)	455.72 (174.23-776.5)	126.62%	118.49%
Oman	11.24 (4.34-19.08)	456.48 (179.19-776.84)	1.57 (0.54-3.02)	42.15 (15.05-76.28)	74.26%	109.31%
Pakistan	5198.29 (2041.57-8452.17)	137034.61 (53296.27-223914.93)	10.71 (4.02-18.45)	228.47 (86.73-385.87)	41.19%	50.09%
Palau	0.73 (0.29-1.21)	17.7 (7.06-29.15)	9.69 (3.53-17.59)	174.53 (63.55-315.98)	55.32%	58.32%
Palestine	21.06 (8.1-36.3)	732.67 (287.32-1192.67)	2.18 (0.8-3.94)	55.56 (21.07-95.5)	33.12%	80.28%
Panama	22.7 (8.34-38.94)	455.35 (166.21-790.22)	0.89 (0.31-1.62)	18.3 (6.53-32.93)	84.40%	65.50%
Papua New Guinea	569.84 (219.72-939.87)	14089.29 (5383.49-23015.62)	31.63 (11.96-54.96)	597.51 (226.75-1036.97)	150.70%	140.31%
Paraguay	59.2 (21.56-100.4)	1294.28 (475.32-2160.63)	2.03 (0.7-3.67)	41.75 (14.65-74.29)	126.30%	120.67%
Peru	73.08 (25.66-137.64)	1689.4 (614.26-3045.98)	0.39 (0.13-0.81)	9.04 (3.01-17.47)	25.42%	18.58%
Philippines	1718.57 (672.97-2794.61)	48987.94 (18961.87-78413.88)	4.78 (1.83-8.15)	114.92 (43.91-193.42)	92.57%	106.62%
Poland	472.69 (185.64-814.79)	12932.65 (5285.14-21830.86)	1.12 (0.43-2)	33.21 (12.58-58.3)	-40.79%	-36.92%

Portugal	227.61 (87.9-391.36)	4170.32 (1609.9-6956.95)	1.31 (0.48-2.32)	29.72 (10.87-51.36)	17.31%	-8.27%
Puerto Rico	37.68 (14.11-65.36)	670.85 (255.22-1150.19)	0.74 (0.26-1.35)	16.25 (5.67-29.19)	67.24%	45.86%
Qatar	3.98 (1.55-6.56)	245.97 (96.71-398.19)	1.85 (0.66-3.39)	48.31 (18.11-84.25)	237.29%	435.65%
Republic of Korea	558.62 (216.82-961.27)	14335.01 (5692.61-24051.13)	1.11 (0.4-2.11)	28.12 (10.3-51.39)	37.69%	43.67%
Republic of Moldova	30.06 (11.49-50.99)	897.93 (341.72-1472.1)	0.91 (0.34-1.62)	28.28 (10.43-49.46)	-65.50%	-61.94%
Romania	397.11 (158.88-657.93)	10383.96 (4171.5-16997.01)	1.89 (0.69-3.22)	54.23 (19.89-91.51)	-52.43%	-45.22%
Russian Federation	1401.76 (544.24-2339.52)	41006.82 (15688.23-68995.4)	1.05 (0.39-1.87)	31.96 (11.75-55.71)	-48.33%	-42.30%
Rwanda	59.23 (22.78-109.21)	2020.18 (786.08-3681.91)	2.13 (0.75-4.16)	55.84 (20.07-106.37)	3.93%	14.35%
Saint Kitts and Nevis	0.32 (0.12-0.54)	7.92 (2.88-13.29)	1.05 (0.36-1.93)	22.1 (7.5-40.52)	60.00%	87.68%
Saint Lucia	1.51 (0.57-2.57)	33.91 (13.03-56.92)	1.18 (0.42-2.2)	25.82 (9.11-47.6)	112.68%	113.54%
Saint Vincent and the Grenadines	0.57 (0.21-0.96)	13.02 (4.79-21.42)	0.79 (0.27-1.46)	17.08 (5.91-31.14)	200.00%	178.80%
Samoa	7.71 (3.07-12.89)	174.54 (69.42-288.71)	12.22 (4.61-21.76)	238.53 (90.06-421.59)	20.85%	20.84%
San Marino	0.15 (0.05-0.27)	4.35 (1.63-7.32)	0.29 (0.09-0.58)	11.06 (3.91-20.65)	-21.05%	-10.68%
Sao Tome and Principe	0.83 (0.3-1.46)	26.93 (9.74-47.48)	1.59 (0.51-3.15)	40.32 (13.23-77.99)	25.76%	52.92%
Saudi Arabia	201.81 (79.83-336.59)	7718.01 (3109.89-12793.37)	2.85 (0.99-5.32)	68.27 (24.5-124.94)	104.88%	198.70%
Senegal	101.87 (38.89-171.14)	3002.11 (1135.65-5001.15)	3.01 (1.03-5.61)	72.2 (25.36-129.09)	25.59%	35.76%
Serbia	285.86 (113.49-464.79)	6597.64 (2605.29-10682.85)	2.97 (1.11-5.12)	71.83 (27.05-121.73)	17.13%	13.25%
Seychelles	1.7 (0.68-2.81)	42.71 (16.9-69.33)	3.18 (1.14-5.69)	71.17 (25.92-125.54)	11.84%	28.99%
Sierra Leone	47.16 (17.99-88.37)	1482.19 (556.38-2665.37)	2.88 (1.01-5.58)	71.34 (26.06-133.59)	2.01%	21.12%
Singapore	16.89 (6.4-28)	477.9 (180.94-789.97)	0.37 (0.13-0.68)	10.11 (3.49-18.8)	-43.36%	-31.18%
Slovakia	59.67 (22.45-99.98)	1747.23 (669.71-2868.71)	1.12 (0.4-2.05)	34.08 (12.26-60.05)	1.67%	5.08%
Slovenia	35.22 (13.55-58.56)	782.28 (293.52-1276.16)	1.21 (0.46-2.16)	31.31 (11.6-55)	-22.15%	-27.57%
Solomon Islands	16.61 (6.57-28.1)	443.34 (177.23-740.69)	13.16 (4.84-23.25)	265.46 (98.94-460.17)	115.43%	112.30%
Somalia	87.76 (27.83-171.15)	3134.06 (1078.82-5903.29)	3.49 (1.08-7.27)	90.78 (30.13-179.45)	78.52%	96.89%
South Africa	907.34 (363.64-1461.55)	27971.34 (11150.09-44985.58)	4.11 (1.58-6.95)	110.19 (42.09-183.79)	47.94%	53.01%

South Asia	89203.11 (34242.14-146419.48)	2006642.47 (761516.79-3303092.77)	13.55 (5.07-22.7)	268.57 (100.8-450.49)	97.41%	79.93%
South Sudan	47.36 (17.69-92.15)	1458.48 (550.97-2755.57)	2.9 (0.97-5.91)	69.95 (23.9-137.78)	2.49%	18.83%
Southeast Asia	17591.52 (6763.02-28798.19)	427798.28 (164692.15-698087.29)	6.08 (2.3-10.01)	128.03 (48.92-210.34)	69.35%	67.48%
Southern Latin America	1229.7 (483.04-2021.84)	24540.21 (9770.72-39759.45)	2.45 (0.93-4.12)	50.72 (19.27-84.04)	32.56%	17.30%
Southern Sub-Saharan Africa	1103.74 (438.39-1779.93)	34230.73 (13587.35-54826.22)	4.11 (1.58-6.84)	108.6 (41.96-180.07)	48.82%	55.41%
Spain	1419.28 (545.68-2411.1)	23217.21 (9108.4-38590.3)	1.92 (0.7-3.43)	38.44 (14.21-67.96)	2.23%	-14.78%
Sri Lanka	655.85 (225.2-1227.47)	13387.45 (4810.99-24148.34)	5.19 (1.69-10.2)	96.15 (32.19-185.75)	37.46%	26.45%
Sudan	242.81 (90.98-450.6)	7650.38 (2947.05-13681.38)	2.75 (0.96-5.41)	69.54 (24.85-131.5)	34.74%	46.66%
Suriname	5.22 (1.87-9.1)	129.06 (46.44-218.63)	1.59 (0.55-3.06)	37.32 (13.17-68.74)	65.19%	72.29%
Sweden	66.92 (24.72-115.02)	1688 (621.13-2826.28)	0.48 (0.17-0.88)	14.79 (5.39-26.64)	13.42%	-5.89%
Switzerland	69.02 (27.14-119.52)	1753.3 (683.54-3045.46)	0.6 (0.21-1.09)	18.18 (6.58-32.97)	-22.56%	-21.29%
Syrian Arab Republic	184.9 (74.68-313.44)	5706.57 (2247.53-9540.76)	3.4 (1.21-6.2)	83.99 (30.32-149.44)	83.67%	92.76%
Taiwan (Province of China)	588.54 (236.57-959.89)	11368.01 (4486.59-18467.34)	2.32 (0.87-4.07)	47.55 (17.52-82.89)	125.59%	69.81%
Tajikistan	70.13 (27.03-126.8)	2032.92 (795.83-3592.04)	2.93 (1.04-5.51)	66.26 (24.29-119.56)	-30.99%	-19.17%
Thailand	1353.93 (508.68-2367.64)	30436.76 (11774.65-51024.37)	2.26 (0.81-4.21)	51.9 (18.92-92.85)	13.23%	1.30%
Timor-Leste	32.41 (13.14-56.77)	757.21 (314.71-1292.79)	8.87 (3.4-16.31)	175.58 (68.99-315.42)	201.21%	162.47%
Togo	29.52 (10.75-53.43)	1179.81 (442.99-2022.09)	1.67 (0.56-3.19)	49.11 (17.41-89.33)	92.56%	122.46%
Tokelau	0.06 (0.02-0.11)	1.22 (0.44-2.1)	7.63 (2.56-14.97)	148.69 (51.24-280.7)	-25.00%	-25.15%
Tonga	3.54 (1.38-5.8)	72.77 (28.61-119.32)	8.61 (3.12-15.58)	169.52 (62.17-300.83)	24.65%	9.41%
Trinidad and Tobago	11.16 (3.97-19.49)	272.37 (97.04-460.73)	1.07 (0.37-1.96)	25.6 (8.99-45.79)	62.68%	70.33%
Tropical Latin America	3172.4 (1225.38-5265.27)	71521.74 (28143.75-119572.88)	2.32 (0.88-4.05)	50.83 (19.4-88.19)	6.96%	5.05%
Tunisia	164.78 (57.38-307.02)	5020.68 (1943.1-8508.58)	2.6 (0.83-5.37)	71.32 (25.79-129.56)	134.90%	124.49%
Turkmenistan	29.14 (11.15-48.94)	933.13 (361-1558.98)	1.51 (0.56-2.61)	41.44 (15.44-70.58)	-47.64%	-33.33%
Tuvalu	0.48 (0.19-0.81)	11.48 (4.59-19.13)	10.88 (3.92-19.95)	218.77 (80.11-395.47)	-4.00%	-10.59%
T 眉 rkiye	3163.31 (1150.42-5307.22)	68084.82 (25425.64-113030.9)	6.97 (2.48-12.47)	139.2 (50.06-244.1)	93.90%	82.24%

Uganda	95.92 (37.71-182.63)	3005.5 (1203.51-5615.38)	1.53 (0.53-3.12)	37.37 (13.42-73.2)	22.79%	40.99%
Ukraine	397.29 (146.25-701.39)	11719.18 (4408.29-19675.1)	0.91 (0.32-1.66)	28.27 (10.37-50.15)	-85.95%	-81.53%
United Arab Emirates	35.9 (14.09-59.5)	2000.58 (783.46-3306.58)	3.85 (1.22-7.33)	88.03 (31.59-160.04)	234.89%	395.08%
United Kingdom	720.17 (283.97-1222.95)	18812.55 (7518.03-31541.24)	0.96 (0.36-1.71)	28.58 (10.83-50.62)	-39.39%	-35.06%
United Republic of Tanzania	166.53 (60.33-288.68)	5562.07 (2024.4-9403.24)	1.46 (0.49-2.8)	38.66 (13.61-71.52)	20.88%	35.76%
United States Virgin Islands	0.49 (0.19-0.86)	11.7 (4.5-20.3)	0.51 (0.16-1.01)	12 (4.05-22.82)	53.13%	45.34%
United States of America	4633.76 (1805.04-7646.7)	126610.63 (49040.79-207278.34)	1.37 (0.51-2.4)	40.22 (15.21-69.31)	43.00%	31.74%
Uruguay	95.74 (38-158.51)	1809.85 (713.18-3008.82)	2.78 (1.03-4.86)	59.82 (22.32-103.44)	14.54%	0.43%
Uzbekistan	118 (45.26-195.81)	4015.8 (1569.13-6476.1)	1.08 (0.4-1.88)	29.69 (10.98-51.12)	-19.32%	7.56%
Vanuatu	5.92 (2.29-10.52)	163.16 (63.29-289.6)	8.85 (3.09-17.11)	182.65 (64.89-348.35)	82.15%	80.81%
Venezuela (Bolivarian Republic of)	368.19 (143.68-643.12)	8098.88 (3110.39-13813.32)	2.42 (0.87-4.43)	50.63 (18.32-90.66)	179.91%	159.73%
Viet Nam	2893.77 (1053.62-4753.94)	62623.28 (23616.09-102392.11)	6.64 (2.28-12.2)	127.37 (44.95-226.96)	60.93%	68.86%
Western Europe	6152.44 (2427.21-10259.91)	131535.6 (52351.3-216556.47)	1.02 (0.39-1.72)	26.29 (10.21-44.02)	-7.64%	-14.39%
Western Sub-Saharan Africa	1176.39 (458.96-2003.09)	41232.09 (15941.42-70329.9)	1.35 (0.52-2.32)	36.64 (13.98-62.07)	37.11%	58.33%
Yemen	288.81 (104.69-501.44)	8576.98 (3187.82-14490.25)	4.89 (1.74-9.23)	114.32 (41.44-206.73)	144.73%	143.30%
Zambia	66.27 (23.67-115.69)	2244.54 (825.69-3894.99)	2.19 (0.73-4.17)	56.25 (18.96-104.36)	65.39%	87.79%
Zimbabwe	77.79 (28.68-131.99)	2764.19 (1040.36-4719.03)	2.69 (0.95-5.06)	72.73 (27.16-131.7)	65.86%	83.98%

SDI - socio-demographic index, DALYs - disability-adjusted life years, ASMR - age-standardized mortality rates, ASDR - age-standardized disability-adjusted life year rates.

Supplementary Table S6. ARIMA model parameters and their corresponding AIC and BIC for prediction of ASMR and ASDR (per 100,000) attributable to SHS exposure-related COPD over the next 10 years globally.

Measures	Parameters	AIC	BIC	ME	MAE	MAPE	RMSE	Ljung-Box test (Q value)	p-value
Deaths									
Both	ARIMA(1,1,0)	-51.32	-47.02	-0.005	0.073	0.883	0.093	6.415	0.268
Male	ARIMA(0,1,1)	-37.08	-32.78	-0.001	0.095	1.036	0.118	1.161	0.949
Female	ARIMA(1,1,0)	-53.87	-51.00	-0.020	0.071	0.879	0.091	9.450	0.092
DALYs									
Both	ARIMA(1,1,0)	114.38	117.25	-0.277	1.015	0.588	1.370	5.803	0.326
Male	ARIMA(1,1,0)	127.86	132.16	-0.043	1.354	0.782	1.689	1.851	0.869
Female	ARIMA(1,1,0)	114.87	117.73	-0.265	1.035	0.580	1.376	4.583	0.469

ARIMA - autoregressive integrated moving average, AIC - akaike information criterion, BIC - bayesian information criterion, ASMR - age-standardized mortality rates, ASDR - age-standardized disability-adjusted life year rates, SHS - secondhand smoke, COPD - chronic obstructive pulmonary disease.

Supplementary Table S7. Prediction of ASMR and ASDR (per 100,000) for COPD attributable to SHS exposure over the next 10 years according to ARIMA models with 95% confidence intervals in Global

Year	ASMR of Both	ASMR of Male	ASMR of Female	ASDR of Both	ASDR of Male	ASDR of Female
2022	5.8 (5.61-5.99)	6.71 (6.47-6.96)	5.18 (4.99-5.36)	120.22 (117.45-123)	127.85 (124.38-131.33)	114.3 (111.51-117.08)
2023	5.71 (5.33-6.08)	6.56 (6.11-7.01)	5.14 (4.75-5.54)	119.56 (113.55-125.58)	125.73 (119.22-132.24)	113.55 (107.48-119.63)
2024	5.59 (5.02-6.16)	6.41 (5.83-6.99)	5.11 (4.47-5.75)	118.95 (109.17-128.73)	123.29 (113.97-132.62)	112.86 (102.92-122.8)
2025	5.47 (4.72-6.21)	6.26 (5.56-6.95)	5.09 (4.18-5.99)	118.39 (104.47-132.3)	120.68 (108.81-132.55)	112.2 (97.98-126.43)
2026	5.33 (4.41-6.25)	6.11 (5.32-6.9)	5.06 (3.88-6.25)	117.86 (99.54-136.19)	117.96 (103.78-132.14)	111.59 (92.75-130.42)
2027	5.19 (4.11-6.27)	5.96 (5.08-6.83)	5.04 (3.57-6.51)	117.38 (94.44-140.32)	115.18 (98.91-131.45)	111.01 (87.3-134.71)

2028	5.04 (3.81-6.28)	5.8 (4.85-6.75)	5.02 (3.25-6.79)	116.93 (89.23-144.63)	112.37 (94.18-130.55)	110.47 (81.69-139.24)
2029	4.9 (3.52-6.27)	5.65 (4.63-6.68)	5 (2.94-7.07)	116.52 (83.95-149.09)	109.53 (89.58-129.48)	109.95 (75.96-143.95)
2030	4.75 (3.24-6.25)	5.5 (4.41-6.59)	4.99 (2.62-7.35)	116.14 (78.63-153.65)	106.68 (85.1-128.26)	109.47 (70.13-148.82)
2031	4.59 (2.96-6.22)	5.35 (4.2-6.5)	4.97 (2.31-7.64)	115.79 (73.29-158.28)	103.83 (80.72-126.94)	109.02 (64.23-153.81)

ASMR - age-standardized mortality rates, ASDR - age-standardized disability-adjusted life year rates, COPD - chronic obstructive pulmonary disease, SHS - secondhand smoke, ARIMA - autoregressive integrated moving average.
